# Enhanced Transmission at the Calyx of Held Synapse in a Mouse Model for Angelman Syndrome

**DOI:** 10.3389/fncel.2017.00418

**Published:** 2018-01-04

**Authors:** Tiantian Wang, Geeske M. van Woerden, Ype Elgersma, J. Gerard G. Borst

**Affiliations:** ^1^Department of Neuroscience, Erasmus MC, University Medical Center Rotterdam, Rotterdam, Netherlands; ^2^ENCORE Expertise Center for Neurodevelopmental Disorders, Erasmus MC, University Medical Center Rotterdam, Rotterdam, Netherlands

**Keywords:** Ube3a, synaptic transmission, synaptic morphology, juxtacellular recording, short-term plasticity, axon initial segment, mouse model, action potential

## Abstract

The neurodevelopmental disorder Angelman syndrome (AS) is characterized by intellectual disability, motor dysfunction, distinct behavioral aspects, and epilepsy. AS is caused by a loss of the maternally expressed *UBE3A* gene, and many of the symptoms are recapitulated in a *Ube3a* mouse model of this syndrome. At the cellular level, changes in the axon initial segment (AIS) have been reported, and changes in vesicle cycling have indicated the presence of presynaptic deficits. Here we studied the role of UBE3A in the auditory system by recording synaptic transmission at the calyx of Held synapse in the medial nucleus of the trapezoid body (MNTB) through *in vivo* whole cell and juxtacellular recordings. We show that MNTB principal neurons in *Ube3a* mice exhibit a hyperpolarized resting membrane potential, an increased action potential (AP) amplitude and a decreased AP half width. Moreover, both the pre- and postsynaptic AP in the calyx of Held synapse of *Ube3a* mice showed significantly faster recovery from spike depression. An increase in AIS length was observed in the principal MNTB neurons of *Ube3a* mice, providing a possible substrate for these gain-of-function changes. Apart from the effect on APs, we also observed that EPSPs showed decreased short-term synaptic depression (STD) during long sound stimulations in AS mice, and faster recovery from STD following these tones, which is suggestive of a presynaptic gain-of-function. Our findings thus provide *in vivo* evidence that UBE3A plays a critical role in controlling synaptic transmission and excitability at excitatory synapses.

## Introduction

Angelman syndrome (AS) is a severe neurodevelopmental disorder caused by loss of the maternal allele of the *UBE3A* gene. AS has an incidence of 1:25,000 and affects both genders equally (Mertz et al., 2013). The disorder is associated with intellectual disability, profound speech impairment, motor abnormalities, epilepsy, and specific behavioral abnormalities including autism and impaired sleep rhythm (reviewed in Buiting et al., 2016). *UBE3A* encodes the ubiquitin ligase E3A (UBE3A; also, termed E6-associated protein, E6-AP), which is expressed solely from the maternal allele in mature neurons. Ubiquitin ligase E3A covalently attaches polyubiquitin chains to proteins, resulting in either their degradation by the 26S proteasome or an impact on their activity. Although several substrates have been identified, it is not clear to what extent these targets contribute to the AS phenotype.

Heterozygous mice with a maternally inherited *Ube3a* mutation show phenotypes that are similar to AS patients, including motor, cognitive and behavioral abnormalities (Jiang et al., 1998; Huang et al., 2013; Bruinsma et al., 2015; Silva-Santos et al., 2015). The AS mouse model also shows increased propensity for audiogenic seizures (Jiang et al., 1998; Van Woerden et al., 2007; Silva-Santos et al., 2015). At the cellular level pyramidal neurons in hippocampal area CA1 showed a more negative membrane potential and larger action potentials (AP) plus a lower AP threshold (Kaphzan et al., 2011). This was accompanied by an increased length of the axon initial segment (AIS) and increased expression of Nav1.6 voltage-dependent sodium channels, the α1 subunit of Na^+^/K^+^ ATPase (α1-NaKA), and ankyrin-G, an anchoring protein of the AIS. It was hypothesized that the increased sodium channel expression was a homeostatic adaptation to counteract the decrease in excitability due to the more negative resting membrane potential; reduction of α1-NaKA expression indeed resulted in normalization of the sodium channel expression (Kaphzan et al., 2013). In addition to these postsynaptic effects, UBE3A also affects presynaptic signaling. It is enriched in axon terminals (Burette et al., 2017) and UBE3A may be involved in the vesicle cycle, since *Ube3a* mice show a reduction in the strength of inhibitory transmission onto neocortical pyramidal neurons, which was accompanied by a decrease in the number of synaptic vesicles, and a large increase in the number of clathrin-coated vesicles (Wallace et al., 2012). More recently, it was shown that *ube3a* mutants showed endocytosis defects in the *Drosophila* neuromuscular junction leading to an increased number of boutons and increased synaptic depression (Li et al., 2016).

To what extent these changes in functional neuronal properties are present *in vivo* is unknown. The principal aim of this work was to understand the physiological consequences of the *Ube3a* mutation for excitatory synaptic transmission, by studying the role of UBE3A in the auditory pathway. By recording synaptic transmission at the calyx of Held synapse in the medial nucleus of the trapezoid body (MNTB), critical parameters of pre- and postsynaptic function can be assessed *in vivo*. The calyx of Held synapse has a relay function in the auditory brainstem; each principal MNTB neuron is contacted by a single, giant terminal from a globular bushy cell of the contralateral AVCN. The principal neurons provide inhibition that is both well timed and sustained to many other auditory nuclei (Borst and Soria van Hoeve, 2012). Because of its large size, both pre- and postsynaptic activity can be recorded *in vivo* with a single pipette (Guinan and Li, 1990). Through waveform analysis of the characteristic extracellular waveform that can be recorded during juxtacellular recordings, estimates for synaptic delay, synaptic strength and postsynaptic excitability can be conveniently obtained (Lorteije and Borst, 2011). We found that *Ube3a* mice show significantly fewer postsynaptic failures, as well as faster recovery from AP depression and faster recovery from prespike depression. In addition, the *Ube3a* mice also showed reduced short-term synaptic depression during tone stimuli and faster recovery from synaptic depression, providing *in vivo* evidence for changes in excitatory synaptic transmission.

## Materials and Methods

### Animals and Experiments

Experiments were conducted in accordance with the European Communities Council Directive and were approved by the Animal Ethics Committee of the Erasmus MC.

Generation of the *Ube3a^m-/p+^* knock-out mutant (**RRID**: MGI:2181811; referred to as *Ube3a^Exon*3*^* mice), which carries a deletion of exon 3 of isoform 3 of *Ube3a* (ENSMUST00000107537, NM_001033962), has been described previously (Jiang et al., 1998). This line was maintained (>40 generations) in the 129S2/SvPasCrl background (Charles River) by breeding heterozygous *Ube3a^m+/p-^* males with wild-type females. For the electrophysiology experiments, we crossed *Ube3a^m+/p-^* female *Ube3a* mutants with wild-type C57BL/6J (Charles River) males, to generate heterozygous *Ube3a (Ube3a^m-/p+^*) mutants and littermate controls in the F1 hybrid 129S2-C57BL/6 background.

**FIGURE 1:**
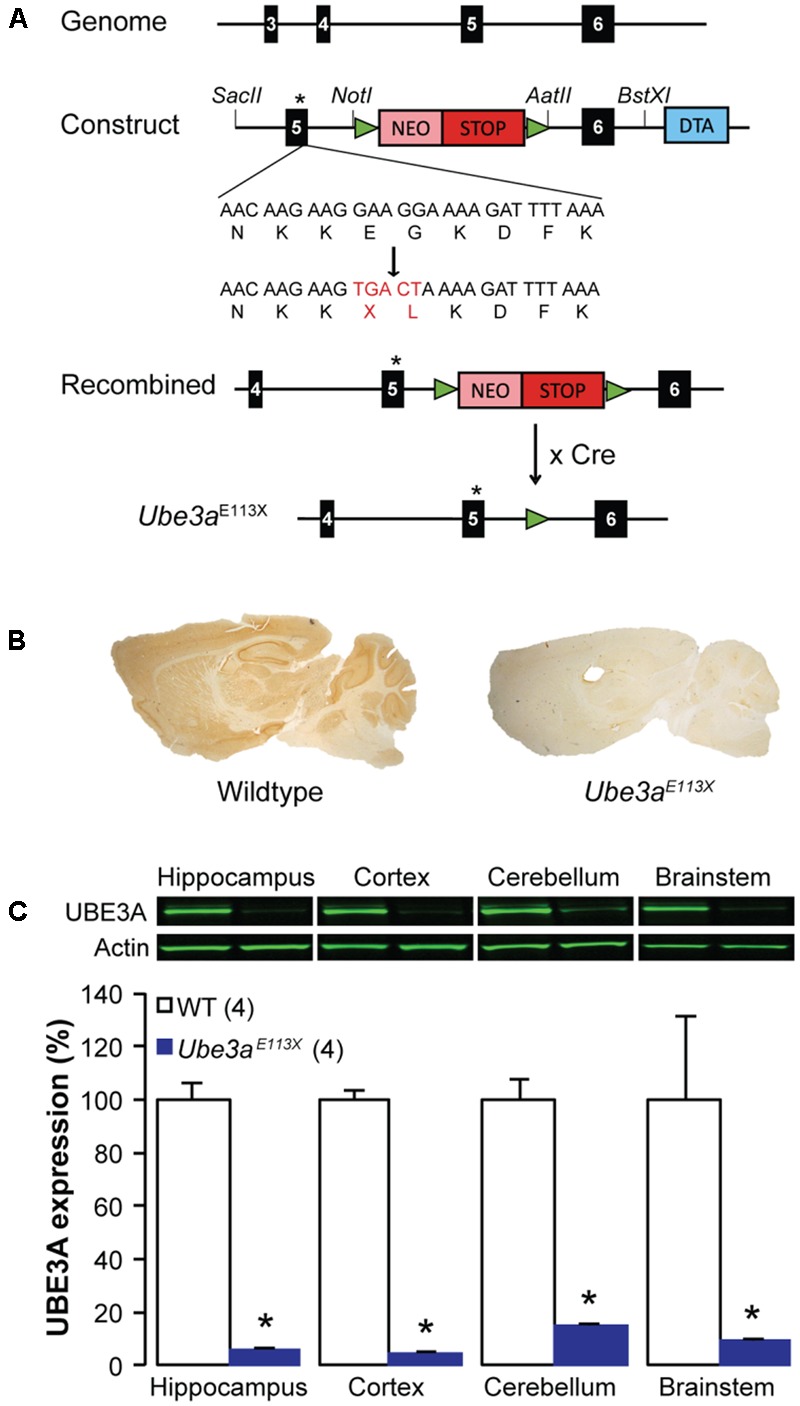
Characterization of the *Ube3a^E*113*X^* mice. **(A)** Schematics depicting the generation of the *Ube3a^E*113*X^* mouse model starting with the insertion of the p.Glu113X and p.Gly114Leu mutations at the end of exon 5, and insertion of a floxed NEO-stop cassette into intron 5–6 of *Ube3a*. Cre-mediated recombination leads to removal of the floxed stop-NEO cassette. Black boxes correspond to *Ube3a* coding exons and green triangles represent the LoxP sites. **(B)** Immunohistochemical UBE3A stainings from a *Ube3a^E*113*X^* mouse and a WT littermate. **(C)** Western blot analysis showing 80–90% reduction of the UBE3A protein in the hippocampus, cortex, cerebellum and brainstem of *Ube3a^E*113*X^* mice compared to WT (*n* = 4 animals for both C57BL/6J WT and *Ube3a^E*113*X^*). ^∗^Indicates *p* < 0.0001 for hippocampus, cortex, cerebellum, and *p* = 0.03 for brainstem.

For the experiments with mice in the C57BL/6J background we used *Ube3a* mutants carrying an E113X nonsense mutation in exon 5 (ENSMUST00000200758.3, NM_011668.2) of the maternally inherited full length (isoform 2) Ube3a gene. This *Ube3a^E*113*X^* mouse mutant (**RRID**: *MGI: 5911277*) was generated as follows (**Figure 1A**): the *Ube3a* genomic sequence (ENSMUSG00000025326) was obtained from Ensembl and used to design the primers for the targeting constructs. PCR fragments encompassing exon 5 using 5′primer: 5′-CCGCGGGCTCCACTAGTCAATTTC-3′ and 3′primer: 5′-GCGGCCGCACCACAGTCCCTGGAGTTC-3′ (4.9 kb; exon denotation according to ENSMUSG00000025326) and exon 6 using 5′ primer: 5′-GGCCGGCCGGAACTACCATATCCTGTTTTAC-3′ and 3′ primer: 5′-GCGGCCGCAGCCGATCTAGGTATTC′ (4.6 kb) were amplified using High Fidelity Taq Polymerase (Roche) on ES cell genomic DNA. Exon 5 and 6 were sequenced to verify that no mutations were introduced. To obtain a premature stop mutation at the end of exon 5 (p.Glu113Stop followed by a p.Gly114Leu mutation) the QuickChange site-directed mutagenesis kit (Stratagene) was used using 5′ primer: 5′-GATAAAAATGAACAAGAAGTGACTAAAAGATTTTAAAGGTAAGAG-3′ and 3′ primer: 5′-CTCTTACCTTTAAAATCTTTTAGTCACTTCTTGTTCATTTTTATC-3′, and the PCR product was sequence verified. Both the 5′ flank with the mutation in exon 5 and the 3′ flank containing exon 6 were cloned on either side of a Neomycin-stop cassette flanked by loxP sites. For counterselection, the diphtheria toxin chain A (DTA) gene was inserted at the 5′ of the targeting construct. The targeting construct was linearized and electroporated into E14 ES cells (derived from 129P2 mice). Cells were cultured in BRL cell-conditioned medium in the presence of leukemia inhibitory factor. After selection with G418 (200 μg/ml), targeted clones were identified by PCR (long-range PCR from neomycin resistance gene to the region flanking the targeted sequence). A clone with verified karyotype was injected into blastocysts of C57BL/6 mice. Male chimeras were crossed with female C57BL/6J mice. The resulting heterozygous offspring were crossed with *TgCAG-cre* mice (**RRID**: *MGI:2176435*) to delete the neomycin gene and stop cassette (**Figure 1A**). *Ube3a^E*113*X^* offspring in which the neomycin cassette was removed and the Cre transgene was absent was used for subsequent crossings with C57BL/6J mice. The *Ube3a^E*113*X^* mice used for this study were crossed minimally 12 times into C57BL/6J.

The *in vivo* juxtacellular recordings were performed in mice of either hybrid 129S2-C57BL/6J background or C57BL/6J background. For the analysis of recovery from presynaptic AP depression and recovery from postsynaptic AP depression, data from both genetic backgrounds were obtained. For the analysis of recovery from EPSP depression, only the data from C57BL/6J background was included, since neurons of hybrid 129S2-C57BL/6J background exhibited very little short-term depression (STD), probably owing to the observed high spontaneous firing rates (**Table 1**). Auditory Brainstem Response (ABR) recordings, *in vivo* whole-cell recordings, slice recordings, immunohistochemistry and quantification of AIS length were performed in *Ube3a^E*113*X^* mice or litter mate controls in the C57BL/6J background.

**Table 1:** Comparison of spontaneous firing and complex waveforms between WT and *Ube3a* mice.

Parameters (juxtacellular)	C57BL/6JWT	*Ube3a*^E*113*X^	*p*-value	Hybrid WT	*Ube3a*^Exon*3*^	*p*-value
Spontaneous failures (%)	10.6 ± 6.4	1.4 ± 1.0	0.026	21.7 ± 5.8	4.0 ± 2.9	0.01
Spontaneous frequency (Hz)	26 ± 6	37 ± 9	0.34	58 ± 4	57 ± 5	0.82
Prespike-eEPSP delay (ms)	0.33 ± 0.01	0.31 ± 0.01	0.41	0.3 ± 0.02	0.34 ± 0.02	0.17
eEPSP-eAP delay (ms)	0.28 ± 0.02	0.23 ± 0.01	0.03	0.34 ± 0.02	0.27 ± 0.01	0.003
eAP halfwidth (ms)	0.24 ± 0.01	0.27 ± 0.01	0.11	0.31 ± 0.01	0.29 ± 0.01	0.21

*Data is from *in vivo* juxtacellular recordings. *Ube3a*^*E*113*X*^ mice: *n* = 14 cells from four animals for C57BL/6J WT, and 15 cells from four animals for *Ube3a. Ube3a*^*Exon*3**^ mice: 20 cells from five animals for 129S2/Sv-C57BL/6J WT, and 21 cells from five animals for *Ube3a*. A *t*-test was used for all the parameters, except for spontaneous failures, for which Mann–Whitney *U* test was used.*

### Auditory Brainstem Responses

Auditory Brainstem Response recordings were performed as described previously (Nagtegaal et al., 2012). Briefly, the mice were anesthetized with a mixture of ketamine/xylazine (60/10 mg/kg) i.p. and placed in a sound-attenuated box in front of a loudspeaker (Radio Shack Super Tweeter 40-1310B), which presented tone pip stimuli (1 ms duration, 0.5 ms cosine-squared ramps, alternating polarity, repetition rate 80 per second) at a sound pressure level (SPL; re 20 μPa) between –10 and 110 dB. Needle electrodes were positioned subdermally at the base of both pinnae; the reference electrode was placed at the vertex, and a ground electrode near the sacrum. For determining ABR thresholds, 500 responses with artifacts <30 μV were averaged. Hearing level thresholds were measured at 4, 8, 16, and 32 kHz. Thresholds were defined as the lowest SPL (5 dB resolution) at which a reproducible peak (usually peak II or IV) was still present in either ear.

### Surgery and *in Vivo* Recordings

After brief exposure to isoflurane, the adult mice (30 to 60 days old) were injected intraperitoneally with a ketamine–xylazine mixture (65 and 10 mg/kg, respectively). A homothermic blanket (FHC, Bowdoinham, ME, United States) was used to keep the rectal body temperature at 36–37°C. Animals were supine positioned, and a tracheotomy was performed so that the animal could be ventilated mechanically with oxygen (MiniVent; type 845; Harvard Apparatus). The animal was ventilated at a frequency of 150/min; with a stroke volume of 7 μl/g body weight. The right anterior inferior cerebellar artery and basilar artery were used as landmarks to locate the right MNTB, as previously described (Rodríguez-Contreras et al., 2008; Crins et al., 2011). Prior to recording, the dura and pia were removed to expose the brain surface. Ringer solution containing (in mM): NaCl 135, KCl 5.4, MgCl_2_ 1, CaCl_2_ 1.8, Hepes 5 (pH 7.2 with NaOH) was applied to keep the brain surface moist.

*In vivo* juxtacellular and whole cell recordings were made from the principal neurons using thick-walled borosilicate glass micropipettes with filament, as described previously (Lorteije et al., 2009). The recording pipette was filled with Ringer solution for juxtacellular recording, and with intracellular solution (K-Gluconate 126, KCL 20, Na_2_-phosphocreatine 10, Mg-ATP 4, Na_2_-GTP 0.3, EGTA 0.5, HEPES 10; pH 7.2 with KOH) for whole cell recording. Potentials were corrected for a junction potential of -11 mV. The recording pipette penetrated the brain surface with a positive pressure of 300 mBar. The pressure was reduced to ∼30 mBar after passing the brain surface, and to 0 mBar when recording started.

To measure the membrane resistance during *in vivo* whole-cell experiments we applied a -10 mV voltage step in whole-cell voltage clamp configuration from a holding potential of -70 mV. Spontaneous events were blanked before averaging (**Figure 5A**). To estimate the membrane resistance, the series resistance, which was calculated from the size of the negative peak at the beginning of the step, was subtracted from the input resistance, which was calculated from the steady-state hyperpolarizing current.

Data were acquired with a MultiClamp 700B patch-clamp amplifier and pCLAMP 9.2 software (MDS Analytical Technologies, **RRID**:*SCR_011323*) at 50 kHz with a 16-bit A/D converter (Digidata 1322A) after filtering at 10 kHz (eight-pole Bessel filter).

### Auditory Stimulation

Closed field sound stimulation was presented as described previously (Tan and Borst, 2007). A speaker probe was inserted into the left ear canal and was stabilized with silicone elastomer. A 2-noise-burst stimulation protocol was designed in MATLAB (Version R2008a), and the auditory stimulus was generated by Tucker Davis Technologies hardware (TDT, system 3, RX6 processor, PA5.1 attenuator, ED1 electrostatic driver, EC1 electrostatic speaker). A MATLAB program controlled both Clampex acquisition and auditory stimulation. The auditory stimulation protocol consisted of a 200 ms silent period, followed by two 400 ms bursts of wide band noise (bandwidth 2–40 kHz; 80 dB SPL), which were presented at six different intervals (40, 80, 160, 320, 640, 1280 ms), followed by a silent period for a total sweep duration of 4 s. Sound intensities were calibrated as previously described (Tan and Borst, 2007). Experiments were performed in a single-walled sound attenuated chamber (Gretch-Ken Industries).

### Preparation of Slices

Adult mice (4–8 weeks old) were anesthetized with isoflurane and decapitated, after which the brain was taken out quickly and submerged in ice-cold carbogenated (95% O_2_, 5% CO_2_) solution containing (in mM): 93 NMDG, 2.5 KCl, 0.5 CaCl_2_, 25 glucose, 10 MgCl_2_, 20 HEPES, 1.25 NaH_2_PO_4_, 30 NaHCO_3_, 2 thiourea, 5 sodium ascorbate, 3 Na-pyruvate, pH 7.4 with HCl (Ting et al., 2014). Coronal slices containing the MNTB were cut in this solution at a thickness of 120 μm using a vibratome (Microm HM 650V, Thermo Scientific). After slicing, the tissue was incubated for 30 min at 37°C in an extracellular medium containing (in mM): 125 NaCl, 2.5 KCl, 1 MgCl_2_, 2 CaCl_2_, 1.25 NaH_2_PO_4_, 0.4 ascorbic acid, 3 myo-inositol, 2 Na-pyruvate, 25 D-glucose, 25 NaHCO_3_ (pH 7.4 in carbogen). Afterward, slices were kept at room temperature until the time of recording.

### Slice Recordings

For recording, slices were placed in the holding chamber of an upright microscope (BX–50, Olympus) and MNTB principal cells were visualized using a 40× (NA 0.8) water immersion objective. For recording of Na^+^ currents, slices were incubated at room temperature, and perfused with a Na^+^-selective solution containing (in mM): 2.5 KCl, 1 MgCl_2_, 2 CaCl_2_, 3 myo-inositol, 2 Na pyruvate, 125 TEACl, 0.1 3,4 diaminopyridine, 25 NaHCO_3_, 25 D-glucose, 0.4 ascorbic acid (pH 7.4). MNTB afferents were stimulated using a bipolar electrode (FHC Inc.) positioned at the midline or half-way between the midline and the MNTB. Whole cell voltage clamp recordings were performed using borosilicate glass pipettes (3–4 MΩ) filled with (in mM): 130 Cs-gluconate, 20 CsCl, 5 Na_2_-phosphocreatine, 4 MgATP, 0.3 Na_2_GTP, 0.5 EGTA, 10 HEPES, pH 7.2. Recordings were performed in whole cell voltage clamp configuration. Whole cell series resistance (<8.5 MΩ) was compensated by 95–98% at a lag of 9 μs. Membrane potentials were corrected on-line for a liquid junction potential of -13 mV. The voltage dependence of recovery from inactivation of sodium channels was tested with a paired-pulse protocol; two 3 ms steps to -10 mV were given at time intervals ranging from 0.2 to 600 ms from a holding potential of -95, -65, or -60 mV.

Stimulus trains were delivered using Clampex 8.2 (MDS Analytical Technologies) protocols via a Digidata 1320A 16-bit A/D converter (MDS Analytical Technologies). Data was acquired using an Axopatch 200B amplifier (MDS Analytical Technologies), filtered at 2–10 kHz with a low pass, four-pole Bessel filter, and sampled at 66.67 or 83.33 kHz with a Digidata 1320A.

### Analysis of *in Vivo* Recordings

Researchers were blinded to genotype until analysis was completed. The analysis was performed using custom procedures written in the NeuroMatic environment (version 2.00, kindly provided by Dr. J. Rothman, University College London, London, United Kingdom; **RRID**:*SCR_004186*) within Igor Pro 6.2 (WaveMetrics, **RRID**:*SCR_000325*).

All *in vivo* recordings reported in this paper showed evidence for the presence of a prespike. Since it was hard to delineate the amplitude of juxtacellular EPSP (eEPSP) from the eAP in many recordings, we used the maximum rate of rise of eEPSP as a measure for the strength of transmission, as previously described (Wang et al., 2013).

To allow measuring the size of prespike at short intervals in juxtacellular recordings, the effects of the previous event on the baseline were subtracted using a custom template procedure. First, complex extracellular waveforms that were not immediately followed by another complex waveform within 2.5 ms were sorted into 4–6 subgroups based on their maximum downward rate and averaged following removal of outliers. For each complex waveform, the averaged waveform was found that had the best matching downward phase; this averaged waveform was aligned with the event at the point during the downward phase where the baseline was intersected (presumably corresponding with the peak of the intracellular AP; Lorteije et al., 2009), scaled, and subtracted from the event to allow a more accurate measurement of the prespike amplitude on the next event (**Figures 8A,B**).

The EPSP amplitudes or maximum rates of rise were fitted with a simple model for short-term plasticity (Varela et al., 1997), as described previously (Crins et al., 2011). In its simplest form, a single depression state parameter decreases at each event with a fraction called the depletion factor (comparable to the release probability of the terminal) and recovers continuously with a single time constant. Synaptic transmission is equal to the product of the depression state parameter and the transmission strength in the absence of short-term plasticity.

We estimated the AP threshold of the MNTB neuron in two different ways. Firstly, because of the difficulty to separate EPSP and AP, we measured the point where the first derivative of EPSP reached its maximum, and took that point as the AP threshold. Secondly, we also measured AP threshold by analysis of subthreshold events in neurons with failures. A plot of the peak membrane potential reached during subthreshold EPSPs against event interval showed a similar distribution of peak membrane potentials at intervals >10 ms, but more positive potentials as the intervals decreased, indicating decreased excitability, due to refractoriness (**Figure 5**). We used the most positive peak potentials at long intervals as an alternative estimate for the AP threshold. This estimate correlated well with the inflection potential (*r* = 0.97), but was on average 5 mV more negative.

Note that we use the term ‘excitability’ as any change that increases the probability that a given synaptic conductance may trigger an AP, including a change in recovery from spike depression.

### Analysis of Sodium Currents in Slice Recordings

The amplitudes of Na^+^ currents were measured as the difference between peak and local baseline using a custom-written Igor procedure. The start and end positions of the local baseline were defined by the user.

### Morphology of the Calyx

Afferent fibers to the calyx of Held were electroporated with Alexa Fluor 594-labeled dextrans (10,000 MW; Invitrogen, Cat# D22913) at the midline *in vivo* in young adult mice (P30–P60) as described previously (Rodríguez-Contreras et al., 2008). One hour later, the animal was perfused and the brainstem was sliced into 40-μm-thick sections. Postsynaptic cells were stained with SYTOX Blue (1:1000; Invitrogen, Cat# S11348).

A laser scanning confocal microscope (LSM 700; Zeiss) equipped with krypton-argon and helium-neon lasers was used to acquire high resolution *z*-stack images (0.5 μm steps; 63X oil immersion objective, NA 1.4) of randomly selected calyces of Held using optimized laser power, detector gain, and pinhole diameter settings. Signal-to-noise ratio was defined as previously described (Di Guilmi et al., 2014), and was at least 20. The number of swellings per calyx was counted on 3D-rendered (Volocity 4.2; Improvision, **RRID**: *SCR_002668*) images of calyces with adjusted contrast and brightness. The surface area and volume of the calyces were measured using the region-of-interest function in Volocity, images of calyx terminals were binary thresholded using the built-in thresholding function of ImageJ 1.46 (isodata algorithm, **RRID**: *SCR_003070*).

### Immunohistochemistry

After recording, mice were perfused transcardially and the brains were post-fixed with 4% paraformaldehyde in sodium phosphate buffer (PB) for 1 h. After sinking in 10 and 30% sucrose (in 0.1 M PB), coronal brainstem sections were cut on a sliding microtome (SM2000R; Leica Microsystems, Rijswijk, Netherlands) at a thickness of 40 μm. The brain sections were washed in Tris-buffered saline (TBS; pH 7.6) and were incubated for 1 h in blocking buffer containing 10% horse serum, 0.5% Triton X-100 in TBS. Subsequently, sections were incubated for 48–72 h in TBS containing primary antibodies, 2% horse serum, and 0.4% Triton. The following primary antibodies were used: mouse anti-ankyrin-G (NeuroMab, clone N106/36, Cat# 75-146, **RRID**: *AB_10673030*; 1:1000 dilution), rabbit anti-Nav1.6 (Alomone labs, Cat# ASC-009, **RRID**: *AB_2040202*; 1:100), guinea pig anti-VGLUT1 and 2 (Chemicon; 1:1000 dilution), rabbit anti-CASPR2 (Chemicon; 1:500). After incubation with primary antibodies, the sections were washed in TBS, and transferred into TBS containing 2% horse serum, 0.4% Triton X-100, and 1:1000 dilutions of Alexa Fluor-594 labeled anti-mouse antibody (Invitrogen, Cat# A-11005, **RRID**: *AB_141372*), Alexa Fluor-633 labeled anti-rabbit antibody (Invitrogen, Cat# A-21070, **RRID**: *AB_2535731*), Alexa-488 labeled anti-guinea pig antibody (Invitrogen, Cat# A-11073 **RRID**: *AB_142018*). After a 2-h incubation at room temperature, the sections were again washed with TBS and 0.1 M PB, followed by a 1-min staining with Sytox Blue (Invitrogen). Afterward, sections were washed with 0.1 M PB, mounted on cover slides and covered with Vectashield (Vector Laboratories, Peterborough, United Kingdom). Confocal images were acquired with an LSM 700 (Carl Zeiss, Sliedrecht, Netherlands).

For UBE3A immunohistochemistry, procedures were the same as above, except brains were embedded in a sucrose/gelatin mixture (10 and 12%, respectively), incubation with primary antibody (mouse anti-E6AP, E8655 Sigma–Aldrich; 1:2,000, **RRID**: *AB_261956*) was overnight, the secondary antibody (anti-mouse HRP, P0447 Dako; 1:200, **RRID**:*AB_2617137*) was detected by 3,3′-diaminobenzidine (DAB) as the chromogen, and DAB sections were analyzed and photographed using a Leica DM-RB microscope and a Leica DFC450 digital camera.

### Quantification of AIS Length

Ankyrin-G immunosignal, which appeared to be evenly distributed throughout the AIS, was used in the quantification of AIS length. The immunosignal of Vglut1 and CASPR2 were used to identify the calyx of Held and nodes of Ranvier, respectively. High resolution *z*-stack confocal images were acquired as described above. The *z*-stack images of initial segments were thresholded using the 3D Hysteresis Thresholding ImageJ plugin. The lower threshold was determined by thresholding a single plane in the middle of the image stacks with the IsoData algorithm. The higher threshold was set at twice the lower threshold. Thresholded image stacks were then loaded in Volocity, and the length of initial segment was quantified by applying the skeletal length measurement to the 3D-rendered structures.

### Western Blot Analysis

To collect tissue for Western blot analysis, brain tissue was dissected from adult mice and immediately frozen in liquid nitrogen. The lysates were prepared by homogenization in lysis buffer (10 mM Tris-HCL pH 6.8, 2.5% SDS) supplemented with protease inhibitor cocktail (P8340, Sigma–Aldrich). After centrifugation (6000 rpm for 5 min) supernatants were collected and concentration was measured using the Pierce BCA protein assay kit (#23225, ThermoFisher Scientific). A total of 20 μg of each sample was loaded on the gel and a wet transfer was performed. The blotted nitrocellulose membrane was probed with antibodies directed against E6AP (E8655 Sigma–Aldrich; 1:1,000, **RRID**: *AB_261956*) and Actin (MAB1501R Millipore; 1:20,000, **RRID**: *AB_2223041*). A fluorophore-conjugated secondary Goat anti-mouse antibody (Westburg, IRDye 800CW; 1:15,000, **RRID**: *AB_2687825*) was used and the protein was detected using Li-cor Odyssey Scanner system. Quantification was done using Odyssey 3.0 software (Li-cor Biosciences). Number of animals used for quantification was 4 for each genotype.

### Statistical Analysis

Data is presented as the mean ± standard error of the mean (SEM). Statistical significance of differences between means was assessed using Student’s *t*-test or Mann–Whitney *U* test if data were not normally distributed. No correction for multiple testing was applied.

## Results

### Generation of *Ube3a* Mice

*Ube3a* mutants carrying an E113X nonsense mutation in exon 5 were generated and crossed into C57BL/6J as described in the Section “Materials and Methods” and **Figure 1A**. Immunohistochemistry showed a strong reduction of UBE3A protein throughout the brain (**Figure 1B**). This reduction was quantified by Western blot analysis of the cortex, hippocampus and brainstem, showing that mice with a maternally inherited *Ube3a^E*113*X^* mutation had a 80–90% reduction of UBE3A protein (**Figure 1C**). This reduction is similar to the commonly used *Ube3a^Exon*3*^* mice (Jiang et al., 1998).

### Normal Hearing Thresholds in *Ube3a* Mice

**FIGURE 2:**
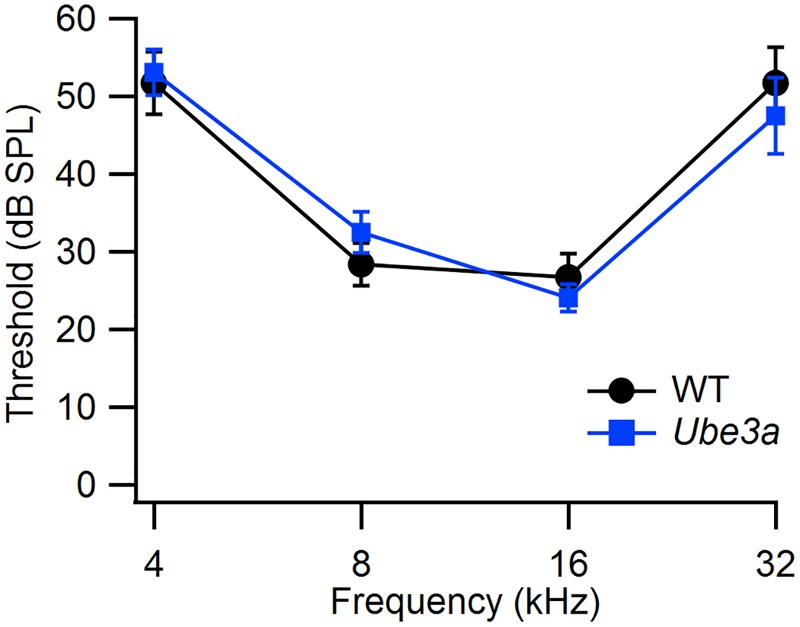
Similar hearing thresholds in WT and *Ube3a* mice. Comparison of the hearing thresholds between WT (*n* = 6) and *Ube3a^E*113*X^* mice (*n* = 11) at four different frequencies.

We used ABR to compare hearing thresholds in *Ube3a^E*113*X^* mice and WT controls in the C57BL/6J background. At the age where we tested the mice, thresholds at 32 kHz were not yet elevated, and WT controls had thresholds that were similar to published values (Zheng et al., 1999). Hearing thresholds of *Ube3a^E*113*X^* mice (*n* = 11 animals) and WT controls (*n* = 6 animals) were similar (**Figure 2**).

### Calyx of Held Has Normal Morphology in *Ube3a* Mice

**FIGURE 3:**
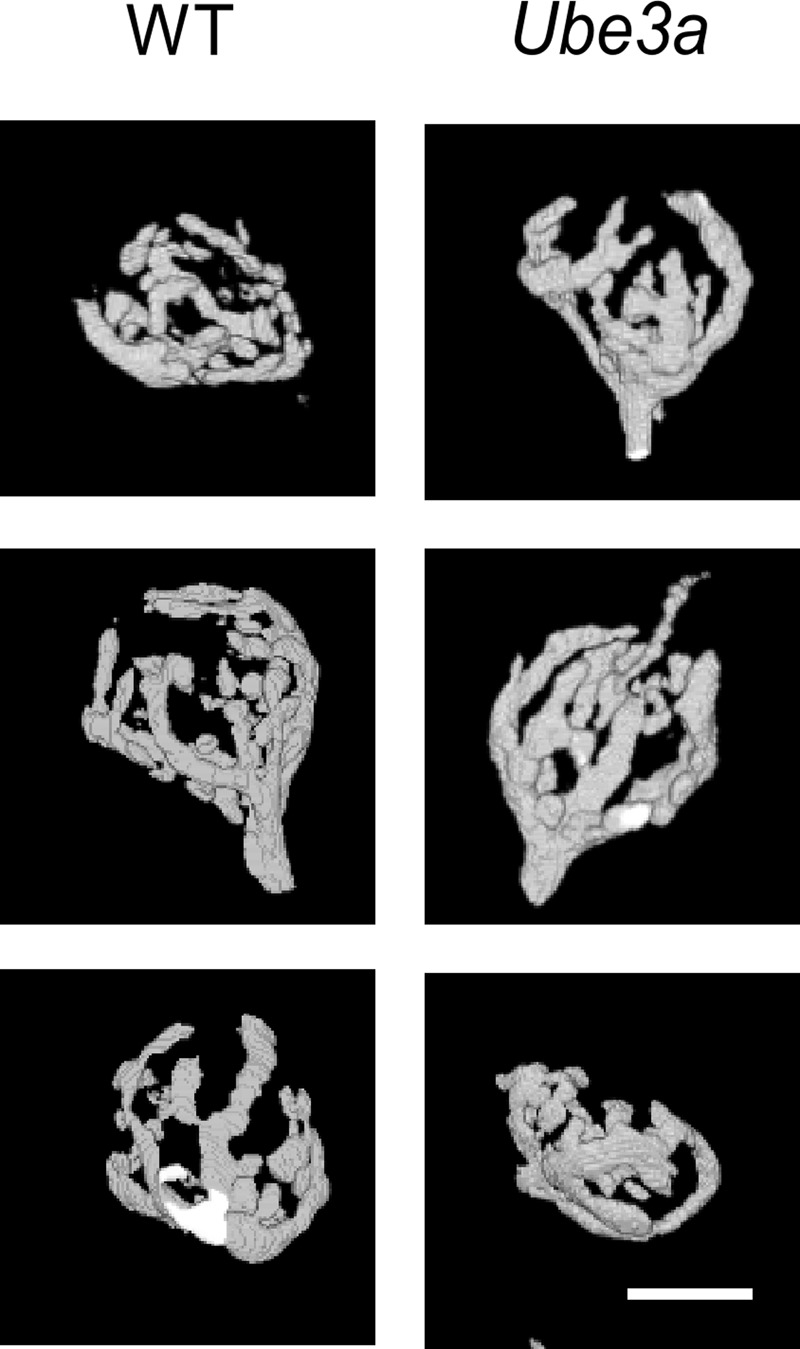
Comparison of morphology of calyx of Held from WT and *Ube3a* mice. Images of 3D-rendered representative calyces from WT (left column) and *Ube3a^E*113*X^* mice (right column). Scale bar, 10 μm.

We compared the morphology of the calyx of Held terminal, which forms a relay synapse in the auditory brainstem, in *Ube3a^E*113*X^* mice and wild-type (WT) littermates in the C57BL/6J background. The mature calyx of Held synapse has a complex structure with fingers containing numerous stalks and swellings (Morest, 1968). Afferent fibers of globular bushy cells (GBCs) were fluorescently labeled *in vivo* by electroporation in P30–P60 animals, followed by confocal imaging of fixed brainstem slices (**Figure 3**). *Z*-stack confocal images were reconstructed in 3D, and the number of boutons, surface area as well as the volume of the calyx was compared between *Ube3a^E*113*X^* and WT. Fifteen *Ube3a^E*113*X^* calyces (from five animals) and six WT calyces (from two animals) were reconstructed. All calyces had swellings originating from thin necks of third order branches. We classified the calyces into three groups according to a previous morphological study (Grande and Wang, 2011): terminals in group I had < 6 swellings, group II had 6–14, and group III had at least 15 swellings. In WT calyces, 3 of 6 calyces belonged in group II and the remainder in group III. In the KI, 8 of 15 calyces belonged in group II and the remainder in group III. Terminals with 6 or less swellings were not observed (Di Guilmi et al., 2014; Wang et al., 2015). Surface area (443 ± 47 μm^2^ in *Ube3a* vs. 454 ± 53 μm^2^ in WT; *p* = 0.89) and volume of calyx terminals (1225 ± 80 μm^3^ in KI vs. 1286 ± 100 μm^3^ in WT; *p* = 0.67) were also similar between *Ube3a^E*113*X^* mutant and WT calyces. We therefore conclude that the gross morphology of WT and *Ube3a^E*113*X^* terminals was similar.

### Decreased Failure Rate in *Ube3a* Mice

**FIGURE 4:**
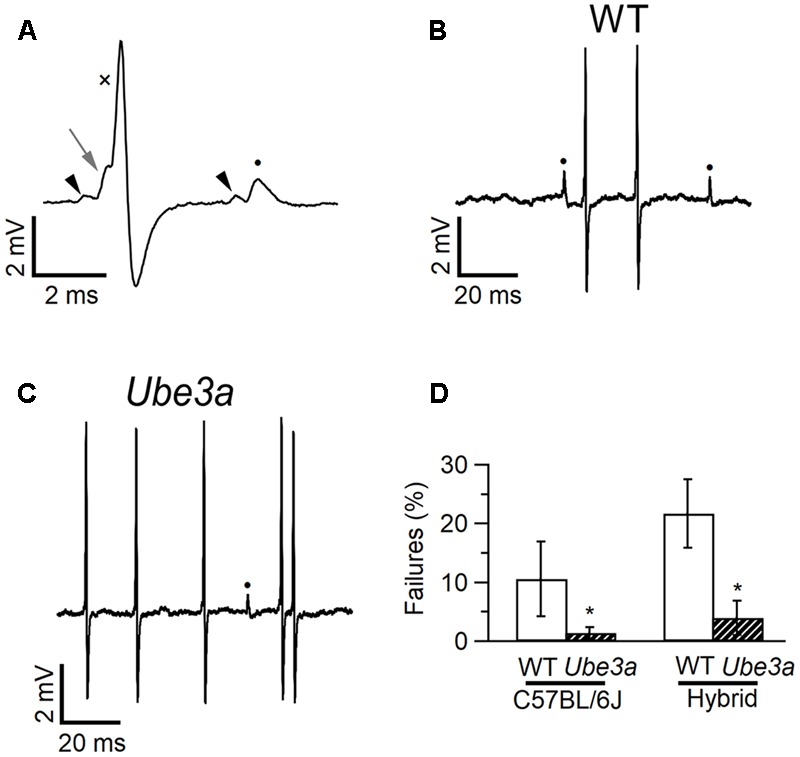
Fewer failures in MNTB neurons of *Ube3a* mice. **(A)** Two complex waveforms from principal neuron of a C57BL/6J WT mouse recorded in the juxtacellular configuration, showing a suprathreshold eEPSP (left gray arrow), which triggers an eAP (cross), and a subthreshold eEPSP (closed circle), which is not followed by an eAP. Both eEPSPs are preceded by a prespike (black arrow heads). **(B)** Same recording as **A**, illustrating the presence of both subthreshold (filled circles) and suprathreshold events. **(C)** Similar as **B**, but from a *Ube3a^E*113*X^* mouse. **(D)** Comparison of spontaneous failure percentages of WT and *Ube3a* mice from two genetic backgrounds. ^∗^Indicates *p* < 0.05 (Mann–Whitney *U* test).

Epilepsy is a common feature of AS (Williams et al., 2006), and audiogenic seizures can be observed in an AS mouse model, but its presence is strongly dependent on genetic background (Van Woerden et al., 2007; Silva-Santos et al., 2015). To investigate changes in excitability and synaptic transmission in the auditory pathway in AS mice, we studied the *in vivo* synaptic activity of the calyx of Held synapse. The recordings were performed in the juxtacellular (loose-patch) configuration, in two independent *Ube3a* lines: the traditionally used *Ube3a* knock-out line in which exon 3 is deleted (*Ube3a^Exon*3*^* mice; Jiang et al., 1998) in the hybrid F1 129/Sv-C57BL/6 background, and a novel *Ube3a* mutant in the C57BL/6 background harboring a E113X nonsense mutation in exon 5 (*Ube3a^E*113*X^* mice). Both lines result in the loss of maternal UBE3A protein expression. The identification of single-unit recordings from principal neurons was based on their characteristic complex waveform (Guinan and Li, 1990). The complex waveforms consist of a small prespike and a larger, brief, positive deflection, which have previously been shown to originate from the calyx of Held and the principal cell, respectively (Lorteije et al., 2009). The good signal-to-noise ratio obtained with the juxtacellular (loose-patch) configuration allowed a clear discrimination between suprathreshold and subthreshold synaptic events (**Figure 4A**). The average spontaneous firing frequency was on average higher in *Ube3a^Exon*3*^* mice in the hybrid 129S2/Sv-C57BL/6 background compared to *Ube3a^E*113*X^* mice in the C57BL/6 background, but these frequencies were similar compared to their respective wild-type littermates (**Table 1**). The percentage of subthreshold events was greatly reduced (**Figures 4B,C**). Wild-type mice in the C57BL/6J background showed postsynaptic failures in 10 of 14 cells, and the average failure rate was 10.6 ± 6.4%. In contrast, in *Ube3a^E*113*X^* mice in the C57BL/6J background only 4 out of 15 cells exhibited failures, and the average percentage of failures was only 1.4 ± 1.0% (*p* = 0.026, Mann–Whitney *U* test). Moreover, we observed a similar significant decrease in spontaneous failures in the *Ube3a^Exon*3*^* mice in the hybrid 129S2/Sv-C57BL/6J background (**Figure 4D** and **Table 1**). Given that we observed this phenomenon in two independent *Ube3a* mutants with different genetic backgrounds, these results strongly point toward a critical role of UBE3A in controlling the strength of synaptic transmission.

We next examined the time course and shape of the complex extracellular waveforms (**Table 1**). AS mice exhibited significantly shorter eEPSP-eAP delays compared to WT littermates, whereas prespike-eEPSP delays were not statistically different. This phenotype was again observed in both mutants and genetic backgrounds.

**FIGURE 5:**
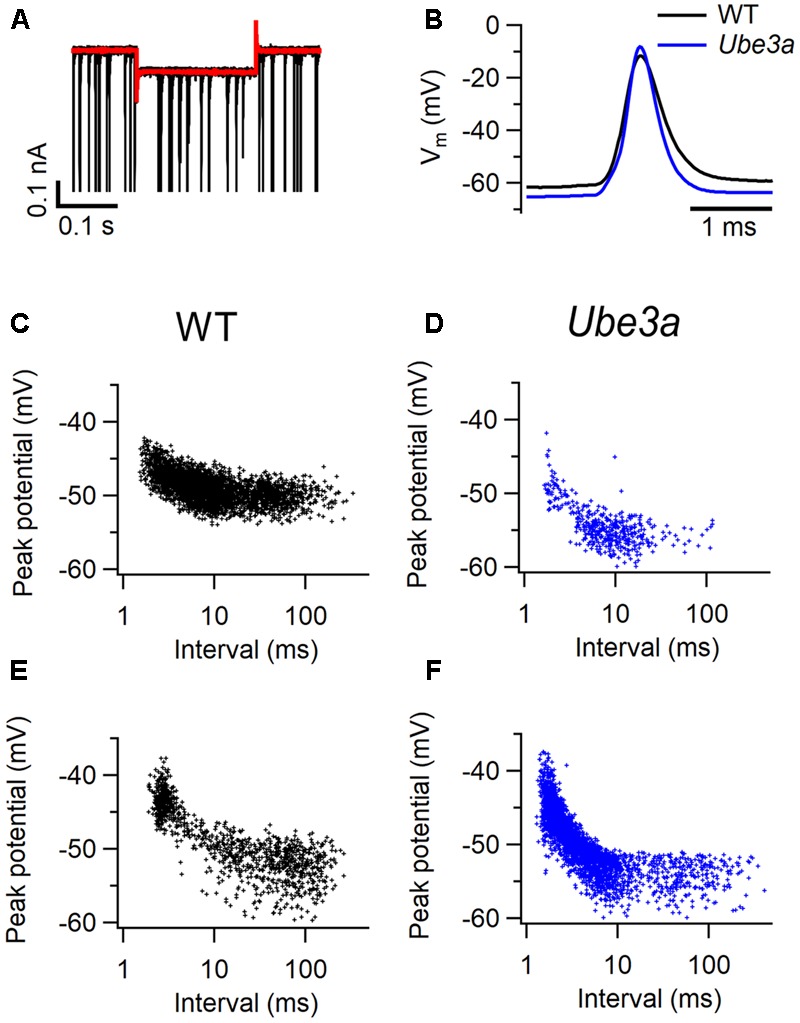
Intrinsic properties of MNTB neurons in *Ube3a* mice. **(A)** Representative superimposed action potentials (APs) from a C57BL/6J WT (black) and a *Ube3a^E*113*X^* (blue) mouse, recorded *in vivo* in the whole-cell configuration. **(B)** Superimposed average trace (red), and the overlay of 5 original traces with spontaneous activity (black) during a 0.2 s, 10 mV hyperpolarizing step from a holding potential of -70 mV. **(C–F)** Relation between most positive potential reached in subthreshold EPSPs and inter-EPSP interval. Data is from two cells from C57BL/6J WT animals **(C,E)** and two cells from *Ube3a^E*113*X^* animals **(D,F)**.

To further investigate the intrinsic properties of MNTB principal neurons in *Ube3a* mice, whole-cell recordings were performed *in vivo* in *Ube3a^E*113*X^* mice with the C57BL/6J background. In voltage clamp mode, a -10 mV hyperpolarizing step was applied (**Figure 5A**), and series resistance as well as membrane resistance were calculated (see “Materials and Methods”). *Ube3a* mice and WT littermates showed similar membrane resistances (**Table 2**). Little or no evidence for the presence of the non-selective, hyperpolarization-activated cation channel *I*_h_ was observed during the hyperpolarizing voltage step, suggesting that *in vivo* little *I*_h_ is active around the resting membrane potential in both *Ube3a* mice and WT littermates (**Figure 5A**). Spontaneous and evoked AP waveforms were recorded in current clamp mode. MNTB neurons of the *Ube3a* mutants exhibited a more hyperpolarized resting membrane potential than wild-type mice (**Table 2**). Moreover, AP amplitude and its maximum rate of rise were larger in *Ube3a* mutants compared to their wild-type littermates, whereas width at half-maximum was shorter in *Ube3a* mutants (**Figure 5B**). Since previous studies showed lower AP threshold in pyramidal neurons of AS mice (Kaphzan et al., 2011), we measured AP threshold of the MNTB neuron in two different ways, as detailed in the Methods. There was no significant difference in the threshold potential between *Ube3a* mice and their wild-type littermates measured based on the inflection point between EPSP and AP or based on failure analysis (**Figures 5C–F** and **Table 2**).

**Table 2:** Comparison of spontaneous firing, complex waveforms and intrinsic properties between WT and *Ube3a^*113*X^* mice.

Parameters (whole-cell)	WT	*Ube3a^E*113*X^*	*p*-value
Spontaneous failures (%)	13.5 ± 7.6	8.5 ± 5.2	0.59
Spontaneous frequency (Hz)	19 ± 5	26 ± 3	0.34
Membrane resistance (MΩ)	72.4 ± 7.1	93.6 ± 7.3	0.06
Resting potential (mV)	-61.8 ± 2.4	-67.2 ± 1.5	0.04
AP amplitude	47.9 ± 2.4	54.9 ± 2.3	0.05
AP dVdt (V/s)	152.3 ± 14.8	203.1 ± 18.1	0.05
AP half width (ms)	0.55 ± 0.04	0.44 ± 0.03	0.03
Inflection point (mV)	-57.6 ± 2.0	-60.2 ± 1.4	0.15
Estimated threshold (mV)	-52.2 ± 2.9	-55.0 ± 2.4	0.6

*Data is from *in vivo* whole-cell recordings from WT (*n* = 8 cells from seven animals) and *Ube3a* (*n* = 9 cells from seven animals). Statistical differences of the mean were assessed by *t*-test.*

### *Ube3a* Mice Show Enhanced Recovery from Postsynaptic AP Depression

**FIGURE 6:**
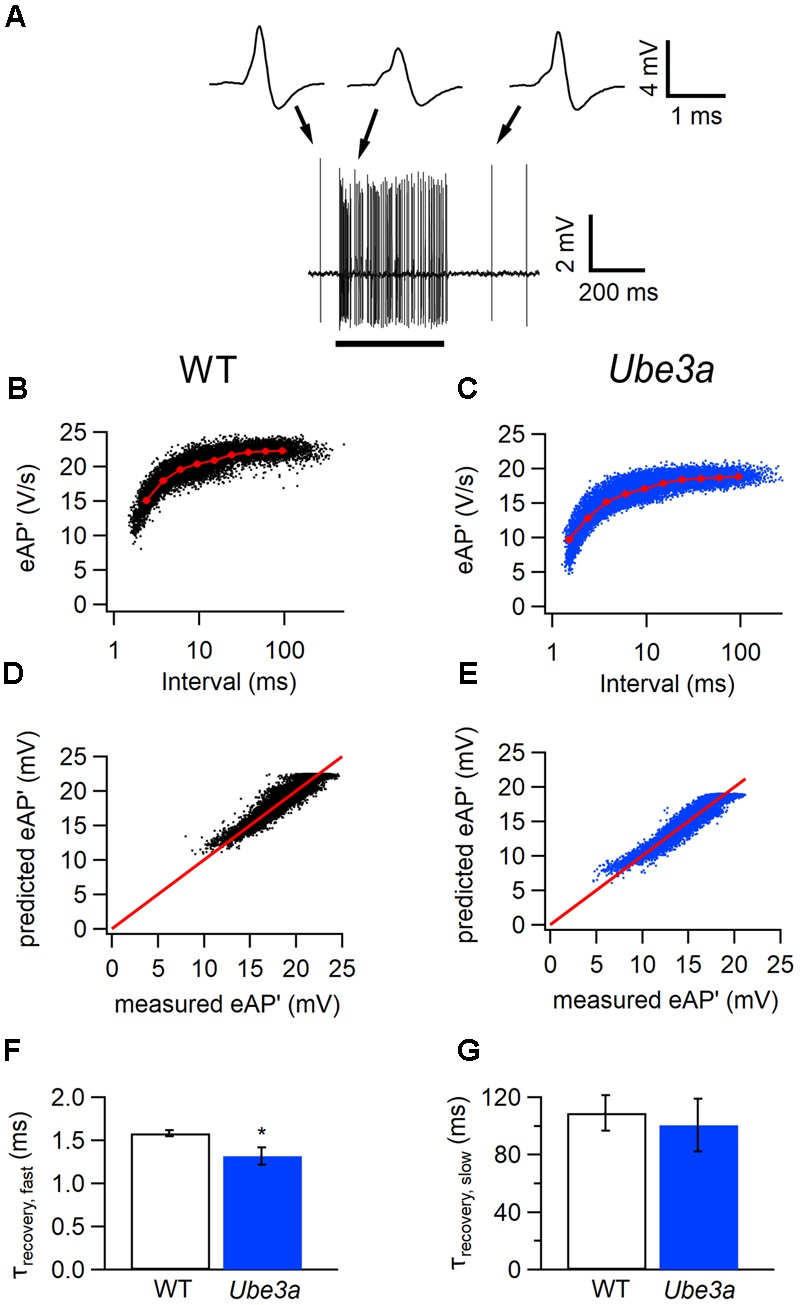
Faster recovery from AP depression in *Ube3a* mice. Recordings were made in the juxtacellular (loose-patch) configuration. **(A)** Increase in spike frequency during a 400 ms, 80 dB noise burst (solid line). Insets are three juxtacellular complex waveforms before, during and after the sound stimulation at higher time resolution. **(B,C)** Relation between the maximal amplitudes of the first derivative of the eAP and the interspike interval in a cell from a C57BL/6J WT **(B)** and a cell from a *Ube3a^E*113*X^* mouse **(C)**. Red line indicates the binned average of the values predicted by the resource depletion model. **(D,E)** Relation between eAP’ predicted by the model and measured eAP’ for the data shown in **D** and **E**, respectively. **(F)** Comparison of the fast recovery time constant from eAP depression in C57BL/6J WT and *Ube3a^E*113*X^* mice. ^∗^Indicates *p* < 0.05. **(G)** Comparison of the slow recovery time constant from eAP depression in C57BL/6J WT and *Ube3a^E*113*X^* mice.

To further investigate the difference in APs between the *Ube3a* mutants and WT mice, we estimated recovery time constants from AP depression. Presentation of a 400 ms, 80 dB noise burst elicited a clear increase in firing rate. The amplitude of juxtacellularly recorded eAP was attenuated during auditory stimulation, to which presumably sodium channel inactivation made a large contribution, and gradually recovered to the original level after the stimulus (**Figure 6A**). To study the recovery of postsynaptic excitability, we measured the maximum of the first derivative of the extracellularly recorded AP (eAP’) at different inter-AP intervals (**Figures 6B,C**). The relation between the size of the eAP’ and inter-AP interval was fitted with a simple resource depletion model (Varela et al., 1997), as previously described (Lorteije et al., 2009). The recovery of eAP’ could be well described by the sum of a fast and a slow time constant (**Figures 6D–G**). The fast time constant of recovery from eAP’ depression was significantly smaller in *Ube3a^E*113*X^* mice than in C57BL/6J WT (τ*_Ube3a_*: 1.32 ± 0.10 ms, *n* = 14 cells from four animals vs. τ_WT_:1.58 ± 0.04 ms, *n* = 15 cells from three animals; *p* = 0.02; **Figures 6B–F**). The slow time constant of recovery did not differ between *Ube3a^E*113*X^* and C57BL/6J WT (τ*_Ube3a_*: 100 ± 18 ms vs. τ_WT_: 109 ± 12 ms; *p* = 0.7; **Figure 6G**). The model fit explained a large part of the variance in eAP’ amplitudes, both in *Ube3a^E*113*X^* mutants and in C57BL/6J WT animals (80.6 ± 2.7% and 80.7 ± 2.4%, respectively).

**FIGURE 7:**
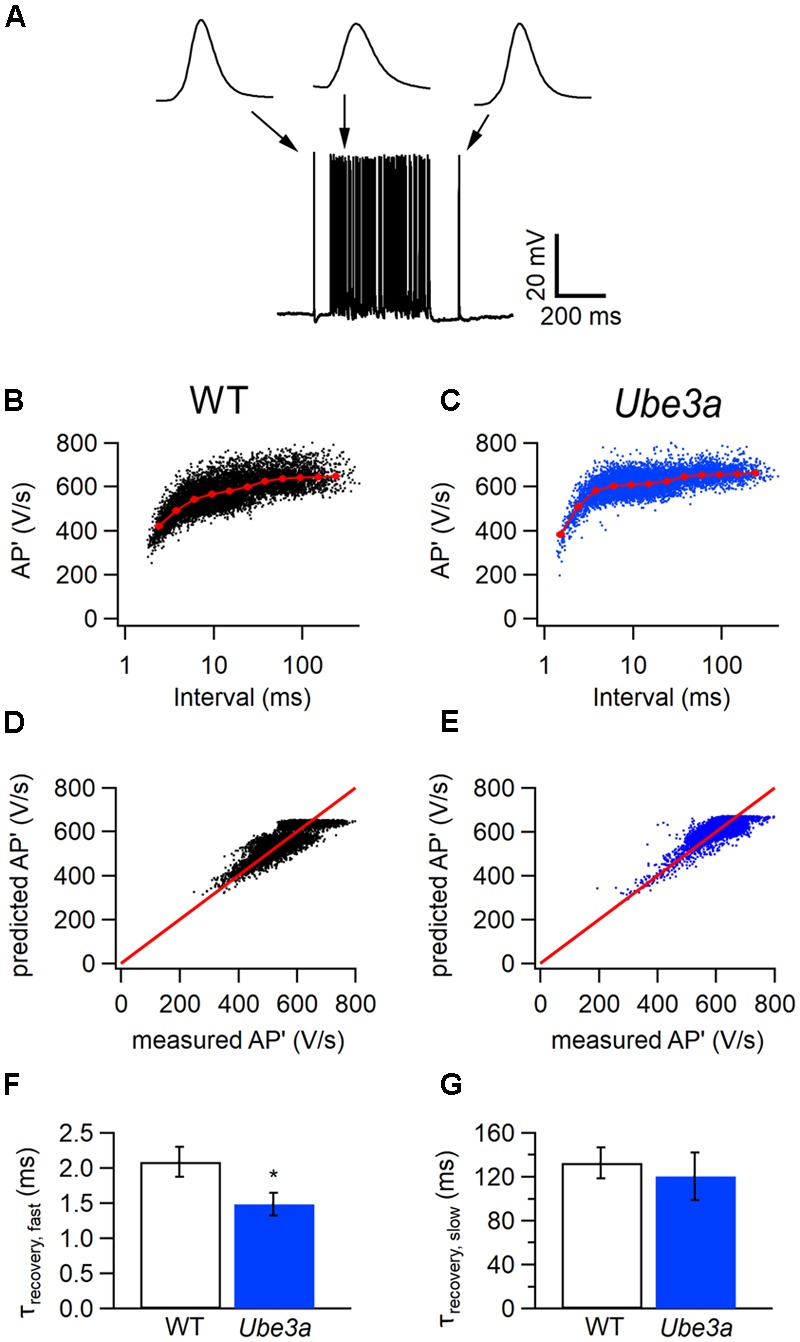
Faster recovery from AP depression in whole-cell configuration in *Ube3a* mice. **(A)** Increase in spike frequency during a 400 ms, 80 dB noise burst in a whole-cell recording. Insets are three principal neuron APs before, during and after the sound stimulation, respectively. **(B,C)** Relation between the maximal amplitudes of the first derivative of the AP (AP’) and the interspike interval from a whole-cell recording (black) from a WT animal **(B)** and a *Ube3a* mouse **(C)**. Red line indicates the binned average of the values predicted by the resource depletion model. **(D,E)** Relation between AP’ predicted by the model and measured in the whole-cell recording for the data shown in **B** and **C**, respectively. **(F)** Comparison of the fast recovery time constant from eAP depression between WT and *Ube3a* mice. ^∗^Indicates *p* < 0.05. **(G)** Comparison of the slow recovery time constant from eAP depression between WT and *Ube3a* mice.

To test whether postsynaptic APs recorded in the whole-cell configuration also recovered more rapidly from depression in *Ube3a* mice, we plotted the maximum of the first derivatives of AP’ against the inter-AP intervals (**Figures 7A–C**). A fit with the resource depletion model showed that the fast time constant for recovery from spike depression was clearly smaller in the *Ube3a^E*113*X^* mice (1.48 ± 0.16 ms, *n* = 8 vs 2.09 ± 0.22 ms, *n* = 6, respectively; *p* = 0.04; **Figures 7B–G**), similar to results obtained in juxtacellular recordings. The faster recovery from AP depression suggests that the kinetics of sodium channel recovery from inactivation are altered in *Ube3a* mice, although a contribution of changes in potassium channel deactivation cannot be excluded.

### *Ube3a* Mice Show Enhanced Recovery from Presynaptic AP Depression

**FIGURE 8:**
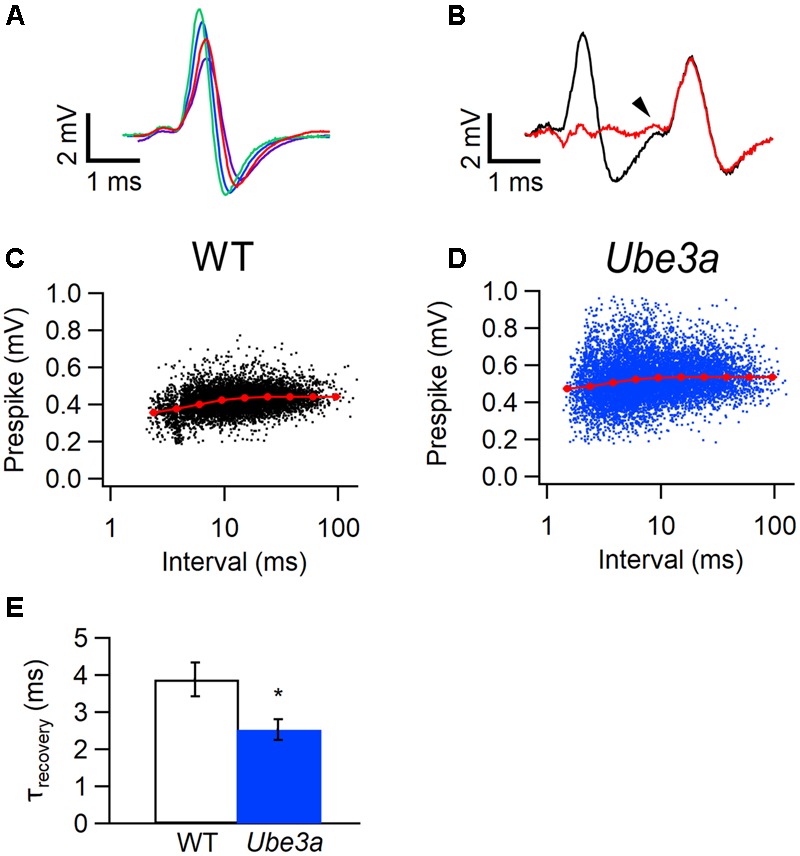
Faster recovery from prespike depression in *Ube3a* mice. Recordings were made in the juxtacellular configuration. **(A)** Four templates used for subtraction; each template was made by averaging complex waveforms with similar amplitudes from a recording from a WT mouse (hybrid background). **(B)** Baseline before (black) and after (red) subtraction of a matching template from the first of the two complex waveforms from the same recording as in A. The prespike is indicated by an arrow head. **(C,D)** Relation between the amplitudes of the juxtacellularly recorded prespikes and inter-EPSP intervals from a WT (**C**, hybrid background) or a *Ube3a^Exon*3*^* animal **(D)**. Red symbols indicate binned averages of the values predicted by the resource depletion model. **(E)** Comparison of the recovery time constant from pre-AP depression between WT and *Ube3a* animals (in both groups three mice with hybrid background and one C57BL/6J mouse). ^∗^Indicates *p* < 0.05.

Next, we tested whether the presynaptic AP were also affected. Information about the calyceal AP can be obtained by measuring the amplitude of the prespikes. When the interval between events is short, the prespike of the next event often falls in the downward deflection of the previous one, making quantification of prespike amplitude difficult. Since the pre- and postsynaptic contribution to the complex extracellular waveform are in principle independent voltage sources, we addressed this problem by subtracting a scaled, averaged waveform from the previous event, as detailed in the Methods and illustrated in **Figures 8A,B**. Only cells with prespikes larger than 0.3 mV were included in this analysis. We plotted the amplitudes of the prespikes against the inter-spike interval, and fitted their relation with a simple model (Varela et al., 1997). The relation between prespike amplitude and the inter-spike interval could be adequately described with a single recovery time constant. Similar to the postsynaptic AP, the prespike of *Ube3a* mice also recovered significantly faster than WT (2.53 ± 0.28 ms, *n* = 4 cells from four animals vs 3.88 ± 0.45 ms, *n* = 4 cells from four animals; *p* = 0.043; **Figures 8C–E**). The presynaptic Na^+^ currents depend on the Na_v_1.6 subunit at the pre-calyceal axonal heminode (Leão et al., 2005; Berret et al., 2016; Sierksma and Borst, 2017), and our results suggest possible changes in the recovery from inactivation of this channel.

### No Changes in Recovery from Inactivation in Postsynaptic Sodium Channels from *Ube3a* Mice

**FIGURE 9:**
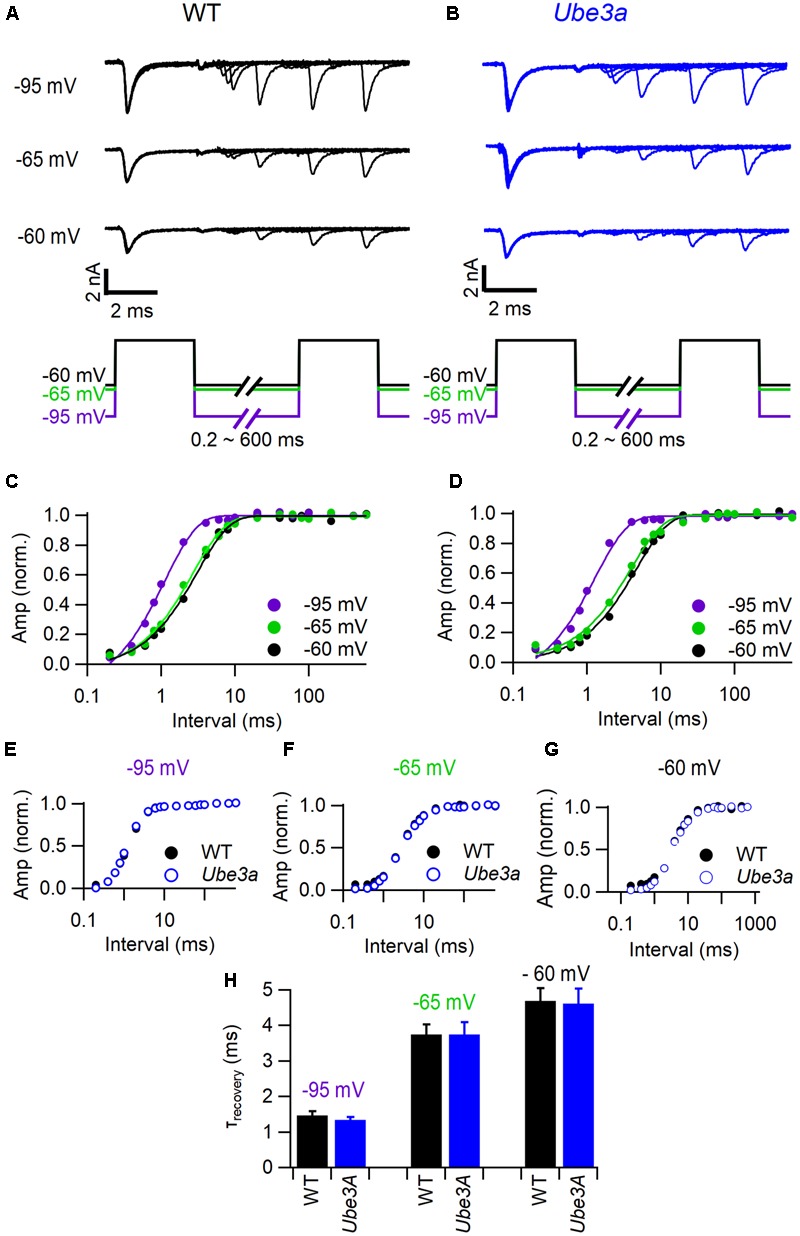
Characterization of Na^+^ current recovery. **(A)** Top panel shows superimposed sodium current recordings from a WT C57BL/6J mouse during two 3 ms depolarizations to -10 mV at time intervals ranging from 0.2 to 600 ms from a holding potential of -95, -65, or -60 mV, respectively; bottom panel shows the stimulation protocol. **(B)** As **A**, but from a *Ube3a^E*113*X^* mouse. **(C)** Dependence of the relative sodium current amplitude on the recovery time interval at a holding potential of -95 mV (purple), -65 mV (green), and -60 mV (black); same cell as **A**; lines indicate mono-exponential fit. **(D)** As **C**, but from the same cell as **B**. **(E)** Average recovery of sodium current at a holding potential of -95 mV (purple) in wild type (filled circles) and *Ube3a* mutant mice (open circles). **(F)** As **E**, except holding potential -65 mV (green). **(G)** As **E**, except holding potential -60 mV (black). **(H)** Comparison of average recovery time constant in different holding potentials between wild type (black) and *Ube3a^E*113*X^* (blue) mice.

To test whether the faster recovery from postsynaptic AP depression in the *Ube3a* mice were caused by a faster recovery from inactivation of voltage-dependent sodium channels, we made whole-cell voltage recordings in slices while pharmacologically isolating the sodium channels. We studied recovery from inactivation with two steps to -10 mV with variable interval at holding potentials of -95, -65, or -60 mV (**Figures 9A,B**). The first step to -10 mV induced a rapid and almost full inactivation. Recovery from inactivation could generally be well described by a single exponential function, whose time constant depended strongly on the potential in between the steps (**Figures 9C,D**). Time constants were in the same range as the fast time constant from AP depression measured *in vivo*, although spatial clamp problems and a difference in temperature (voltage clamp studies were done at room temperature), present some uncertainties. The voltage dependence of sodium channel recovery in WT and *Ube3a* mice was very similar (**Figures 9E–H**), suggesting that the observed difference in AP recovery was not caused by a change in the kinetics of the sodium channels.

### Increased AIS Length of Principal Neurons in the *Ube3a* Mutant

**FIGURE 10:**
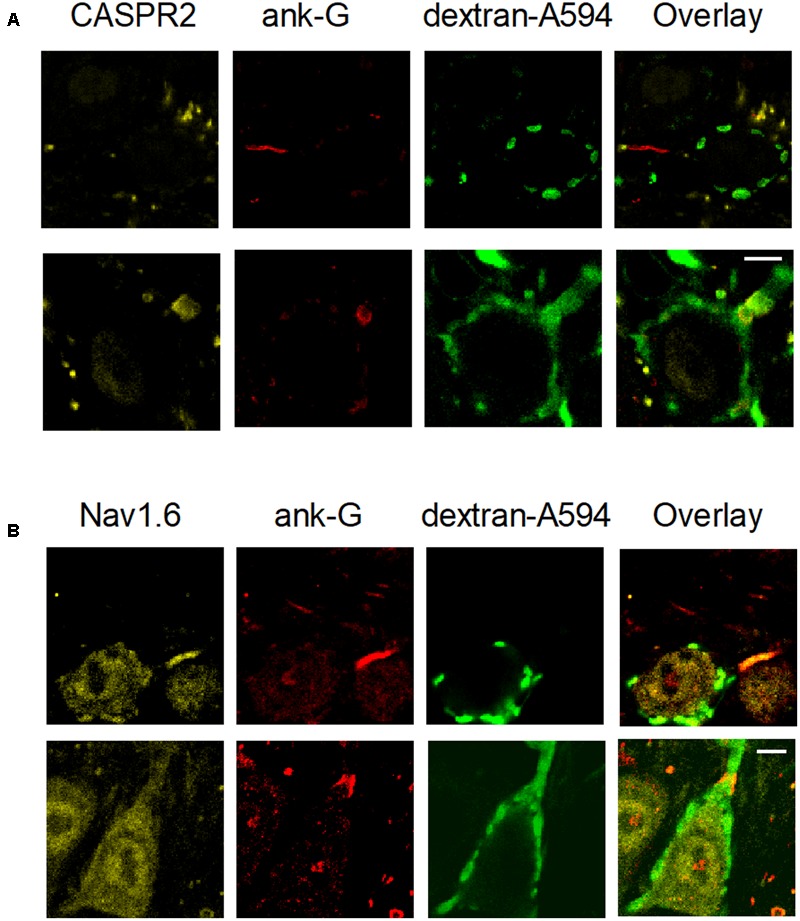
Structural differences between heminodes and axon initial segments (AIS) in the MNTB. Calyces were filled with dextran-A594 via midline electroporation. **(A)** A filled calyx stained with anti-CASPR2 and anti-ankyrin-G antibody, as well as an overlay of these images. **(B)** A filled calyx with staining of anti-Nav1.6 and anti-ankyrin-G antibody, as well as an overlay of these images. Calibration bars 5 μm.

**FIGURE 11:**
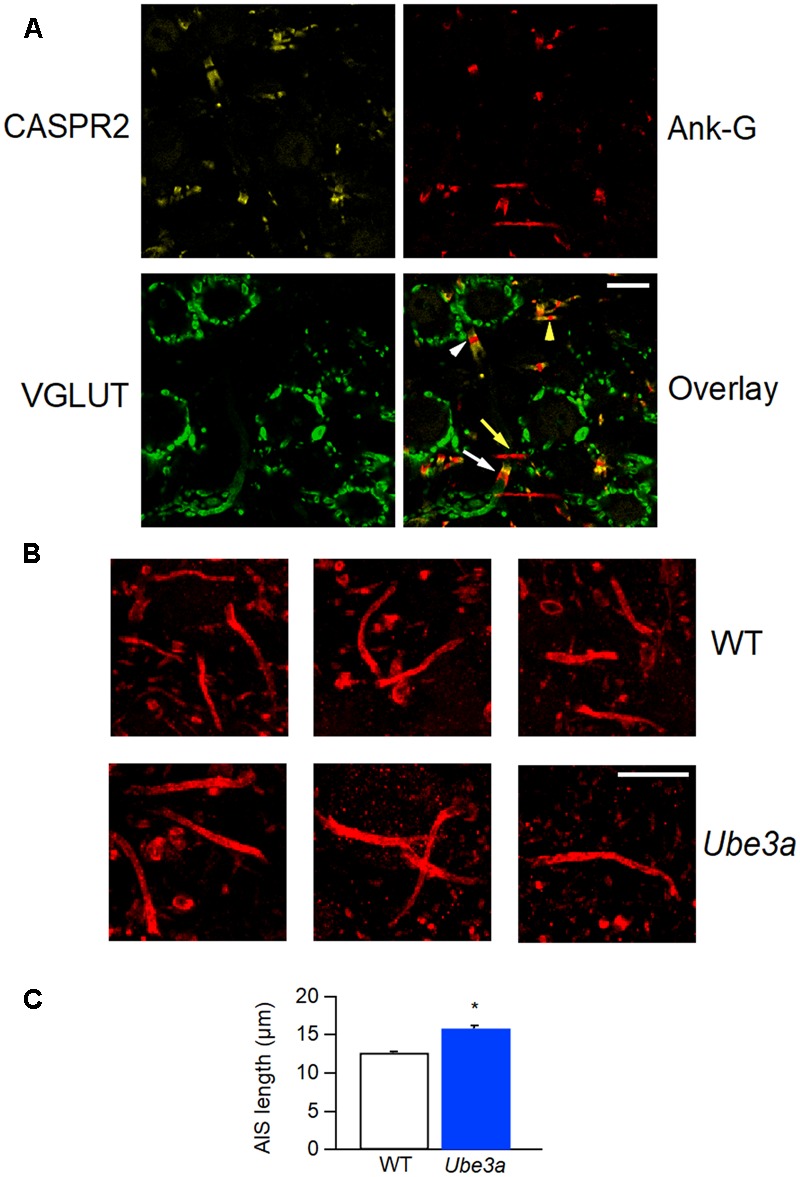
Longer AIS in *Ube3a* mice. **(A)** Confocal section of MNTB slice, labeled with anti-CASPR2 (yellow) and anti-ankyrin-G (red) and anti-Vglut1 & 2 (green). The composite image shows a node of Ranvier from a calyceal axon (white arrow head) and a principal cell axon (yellow arrow head), and a calyceal heminode (white arrow), as well as a postsynaptic AIS (yellow arrow). **(B)** AISs of MNTB cells labeled by anti-ankyrin-G, from both WT and *Ube3a* animals. **(C)** Comparison of AIS length in C57BL/6J WT and *Ube3a^E*113*X^* mice. ^∗^Indicates *p* < 0.001. Calibration bars 10 μm.

Our observations indicate that the *Ube3a* mouse is a gain of function mutant for synaptic transmission in the calyx of Held synapse. Voltage-gated sodium channel Nav1.6 plays a critical role in controlling neuronal excitability and AP generation (Kuba et al., 2014). Its expression level is significantly higher and the length of the AIS is significantly increased in the CA1 and CA3 hippocampal subregions of *Ube3a* mice (Kaphzan et al., 2011). We therefore measured the AIS length of principal neurons in the MNTB using the AIS-organizing protein ankyrin-G as a marker (Kim et al., 2013). Contactin-associated protein 2 (CASPR2), a neurexin protein localized at the juxtaparanodes of myelinated axons (Poliak et al., 1999), was also stained to help discriminate between pre- and postsynaptic axonal structures. AIS immunolabeling of ankyrin-G revealed three types of structures: short structures that were flanked on both sides by CASPR2-positive staining, identifying them as nodes of Ranvier, a thin, bar-like structure that was followed by CASPR2 staining at one end, and a much thicker and shorter structure that always partially co-localized with CASPR2 on one end (**Figure 10A**). To distinguish between the calyceal heminode and the principal cell axons, afferent fibers of GBCs were fluorescently labeled via *in vivo* electroporation, before immunostaining with the combination of anti-ankyrin-G and anti-CASPR2/anti-Na_v_1.6. The thick structures always appeared to be part of the fluorescently labeled fibers before the start of the calyx terminal (**Figure 10**), which indicates these were heminodes (Ford et al., 2015; Xu et al., 2016). In the 758 axons we have measured, the thin, bar-like structures never coincided with fluorescently labeled fibers, and they were always followed by CASPR2 staining at the distal end to the principal neuron, suggesting that the thin bar-like structure is the AIS, which is immediately followed by myelination of principal cell axon. These results are in agreement with the previous finding that the calyceal axon is clearly thicker than the principal cell axon (Forsythe, 1994; Leão et al., 2005). There was also intensive labeling by anti-Na_v_1.6 at the region of AIS (**Figure 10B**), suggesting a significant co-localization of sodium channels and ankyrin-G at the initial segment. Similar to the morphological difference between calyceal heminode and principal cell axon, the presynaptic nodes of Ranvier also appeared thicker than the postsynaptic ones (**Figure 11A**).

Having established that we can reliably identify the AIS, we next quantified the length of the AIS of MNTB cells from both C57BL/6J WT and *Ube3a^E*113*X^* mice. The bar-like ankyrin-G-positive structures from *Ube3a* mice were on average longer than in their WT littermates (12.7 ± 0.2 μm in WT, from 520 AIS of 6 mice vs. 15.9 ± 0.3 μm in *Ube3a* from 238 AIS of 3 mice; *p* < 0.001; **Figures 11B,C**). Assuming a similar density of sodium channels at the AIS of WT and *Ube3a* mice, together with the co-staining results shown in **Figure 11B**, the increased length of the AIS suggests that an increased number of sodium channels are expressed in principal neurons of *Ube3a* mice.

### *Ube3a* Mice Show Diminished Short-term Depression *in Vivo*

**FIGURE 12:**
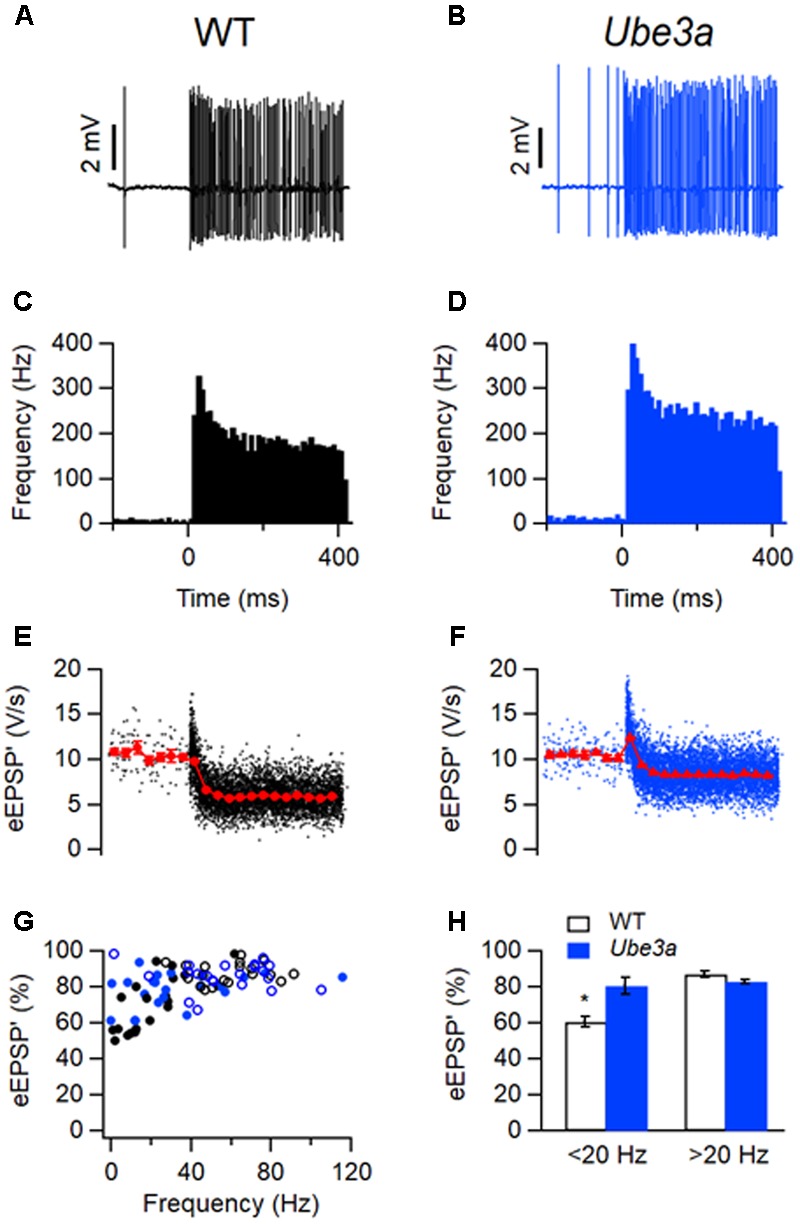
Smaller sound-evoked STD in *Ube3a* mice. **(A)** Increase in frequency during a 400 ms, 80 dB noise burst. **(C)** Peristimulus time histogram showing primary-like response to sound. **(E)** Amplitudes of eEPSP’. Closed circles indicate time-binned averages. Bin size 30 ms. **(A,C,E)** are from the same WT juxtacellular recording from a C57BL/6J mouse. **(B,D,F)** are similar with **(A,C,E)**, respectively, except the recording was from a unit in a *Ube3a^E*113*X^* mouse. **(G)** Relation between eEPSP’ amplitude and spontaneous firing frequency. WT and *Ube3a* animals are indicated in black and blue, respectively. Closed circles and open circles represent cells from C57BL/6J background and 129S2/Sv-C57BL/6J background, respectively. **(H)** Comparison of relative eEPSP’ amplitudes during the last 50 ms of auditory stimulation of cells with a spontaneous frequency <20 Hz and >20 Hz, respectively. ^∗^Indicates *p* < 0.05.

Since the threshold of AP did not change, the lower failure rates of spontaneous synaptic events of AS mice might have resulted from a difference in the calyceal input. For this reason, we studied STD of the first derivative of the EPSP (EPSP’) evoked by sound stimulation. Spontaneous firing in C57BL/6J WT animals averaged 21 ± 4 Hz (*n* = 20; range 1–62 Hz). During auditory stimulation, it reached a maximum frequency of 311 ± 11 Hz within the first 10 ms and decayed to 155 ± 7 Hz during the last 50 ms of the tone (**Figures 12A,C**). Spontaneous firing frequencies in the *Ube3a^E*113*X^* animals averaged 32 ± 6 Hz (*n* = 23; range 0.05–116 Hz). *Ube3a* mutants had similar maximum firing frequencies during the first 10 ms of auditory stimulation, and steady-state frequencies during the last 50 ms as WT animals (**Figures 12B,D**).

We used the maximum of the first derivative of the extracellular EPSP (eEPSP’) or the second derivative of whole-cell EPSP (EPSP”) as a measure for the strength of synaptic transmission, as described previously (Wang et al., 2013). During sound presentation, the average eEPSP’ in both WT and *Ube3a^E*113*X^* mice often decreased to a lower level, indicating that the high firing frequencies induced STD (**Figures 12E–G**). Overall, the *Ube3a^E*113*X^* mutants showed less STD than the WT controls (80 ± 2% of control, *n* = 23 vs. 71 ± 3%, *n* = 20; *p* = 0.04). As previously observed (Wang et al., 2013, 2015), the amount of STD in WT animals depended strongly on spontaneous firing frequencies (**Figure 12H**), presumably because cells with a high spontaneous firing frequency are tonically depressed. In C57BL/6J WT mice, cells with a spontaneous frequency <20 Hz showed large STD (61 ± 3% of control, *n* = 11 cells from five animals). In contrast, STD in *Ube3a* cells exhibited no dependency on spontaneous firing (**Figure 12H**). The *Ube3a^E*113*X^* cells with a spontaneous frequency <20 Hz showed little STD (78 ± 6% of control, *n* = 8 cells from seven animals; *p* = 0.01 vs. WT).

### EPSPs from *Ube3a* Mice Show Faster Recovery from Depression

**FIGURE 13:**
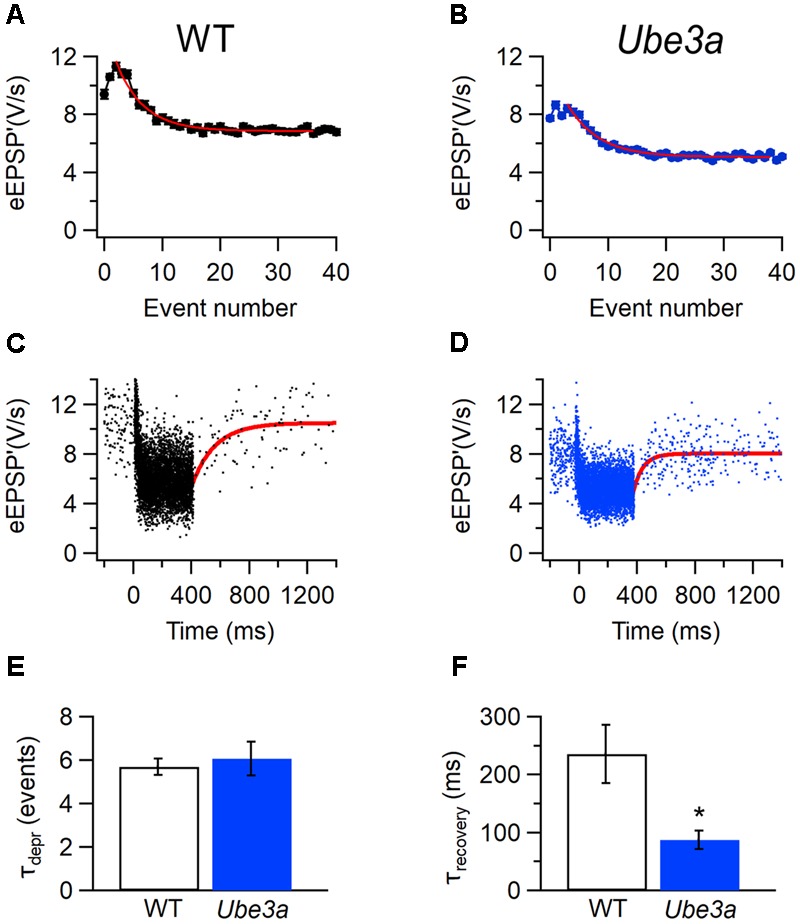
Faster kinetics of recovery from STD in *Ube3a* mice. **(A)** Amplitude of eEPSP’ against sound-evoked event number from recording of C57BL/6J WT cell. Error bars indicate SEM. Solid line is fit with single exponential function. **(C)** Sound-evoked eEPSP’ in a WT animal. Solid line is fit of recovery from sound-evoked STD with single exponential function with time constant 215 ms. **(B,D)** Both are similar with **A** and **C**, respectively, except that the recording was from a *Ube3a* mouse 31 ms. **(E)** Comparison of results of fit of depression time course as shown in A and B for WT and *Ube3a* animal. **(F)** Comparison of recovery time constants as shown in **C** and **D** for WT and *Ube3a*. **A** and **C** are from the same WT recording; **B** and **D** are from the same unit of a *Ube3a* mouse. ^∗^Indicates *p* < 0.01.

To further investigate the difference in STD between *Ube3a^E*113*X^* and WT mice, we estimated the time course of synaptic depression and recovery in both juxtacellular and whole-cell recordings. To quantify how many events it took for the synapse to reach steady-state depression, the relation between the eEPSP’ and its event number was plotted (**Figures 13A,B,E**). The decay of the amplitudes could be well fitted by a single exponential function, as previously described (Wang et al., 2013). During auditory stimulation, *Ube3a^E*113*X^* and C57BL/6J WT took a similar number of events to reach steady state (τ*_Ube3a_*: 6.1 ± 0.7 events, *n* = 22 cells, including eight whole-cell recordings vs. τ_WT_: 5.7 ± 0.4 events, *n* = 20 cells, including six whole-cell recordings; *p* = 0.67). For the estimation of the time course for recovery from STD, eEPSP’ (or EPSP’ in the case of whole-cell recordings) was plotted against time, and fitted with a single exponential function (**Figures 13C,D,F**). In contrast to the lack of an obvious difference in the time course of the onset of depression (**Figures 13A,B,E**), the recovery from depression was overall much faster in *Ube3a^E*113*X^* than C57BL/6J WT cells (τ*_Ube3a_*: 88 ± 14 ms, *n* = 21 cells, including eight whole-cell recordings vs. τ_WT_: 235 ± 51 ms, *n* = 19 cells, including six whole-cell recordings; *p* = 0.006). To confirm these results, we also fitted the data by a short-term plasticity model (Varela et al., 1997), which takes the firing frequency and firing pattern into account. The predicted time constant of recovery from depression was again faster in *Ube3a^*113*X^* than in WT cells (τ*_Ube3a_*: 141 ± 16 ms, *n* = 20 cells including 8 whole-cell recordings vs. τ_WT_: 193 ± 22 ms, *n* = 16 cells including 6 whole-cell recordings; *p* = 0.06), which is in agreement with the direct fits of the recovery after the sound bursts.

## Discussion

Here we show that maternal loss of *Ube3a* leads to enhanced synaptic transmission at the calyx of Held synapse *in vivo.* The *Ube3a* mutant MNTB neurons showed decreased failure rate compared to the wild type. Both pre- and postsynaptic APs recovered faster from depression. These phenotypes were replicated in two independent *Ube3a* mutants in two different genetic backgrounds. In the *in vivo* whole-cell recording of *Ube3a^E*113*X^* mice in the C57BL/6J background, the mutant neurons exhibited altered intrinsic properties, including elevated AP amplitude and decreased AP half width, as well as a more hyperpolarized resting membrane potential. We also observed an increased AIS length in the *Ube3a^E*113*X^* mice in the C57BL/6J background, which might be related to the larger AP. In addition, reduced STD and faster recovery from STD were observed. Taken together, these *in vivo* results imply a critical role of UBE3A in controlling synaptic transmission and excitability at excitatory synapses.

### Synaptic vs. Network Changes

Previous studies of *Ube3a* mice have focused mainly on cortical and hippocampal neurons (Kaphzan et al., 2011; Wallace et al., 2012, 2017). These neurons are innervated by a heterogeneous population of synapses, making it more difficult to distinguish the impact of UBE3A on the many different inputs. In the case of the calyx of Held synapse, synaptic transmission can be studied in relative isolation. Because of the similarity in hearing thresholds and in spontaneous and sound-evoked frequencies in WT and *Ube3a* mice, it is unlikely that the enhanced neurotransmission is caused by upstream effects or an excitatory/inhibitory imbalance within the MNTB, also taking into account that *in vivo* evidence suggests that the direct contribution of inhibitory inputs at the mouse calyx of Held synapse is limited (Lorteije and Borst, 2011). Apparently, the observed increased reliability of excitatory transmission was not sufficient to lead to a substantial increase in spontaneous firing frequency in the MNTB.

### Increased Resistance to Spike Depression

Two main differences in the function of the calyx of Held synapse were observed between *Ube3A* mice and their wild-type littermates. We observed an increased resistance to STD, presumably a presynaptic change in the young-adult calyx of Held synapse, and an increased excitability, probably both pre- and postsynaptically. The two gain-of-function phenotypes are probably unrelated, as the effect on STD acts at about a 100 times slower timescale than some of the effects on excitability. We will first discuss the effects on excitability.

Postsynaptically, we observed a more negative membrane potential, fewer failures, a larger AP and smaller AP half width, and a longer AIS. Both pre- and postsynaptically, we observed faster recovery from AP depression. Several of these findings are most likely related to postsynaptic sodium channels. During high frequency stimulation, AP amplitudes will depress. We used the amplitudes of the juxtacellularly recorded prespike and postspike, which provide a measure for the maximum rate of rise of the pre- and the postsynaptic AP, respectively (Lorteije et al., 2009), to compare AP depression between *Ube3a* and WT. In this study, we observed that APs from *Ube3a* principal MNTB cells recovered faster from depression than in WT. In addition, evidence for faster recovery of calyceal APs *in vivo* was obtained. Recovery of the rate of rise of the AP amplitude can be expected to depend foremost on the recovery of sodium channels from the inactivated state, which is both a time- and a voltage-dependent process (Ming and Wang, 2003).

The recovery from presynaptic spike depression could be described by a single exponential function, in agreement with the mono-exponential recovery from inactivation of sodium channels in calyces (Leão et al., 2005) or hippocampal mossy fiber boutons (Engel and Jonas, 2005). A direct comparison of the kinetics of recovery of the sodium channels and of the spike depression shows that the spikes recovered somewhat more slowly, which may be related to the strong voltage-dependence of the fast component of the recovery from sodium channel inactivation (Leão et al., 2005; Mathews, 2008). In addition, the small size of the prespikes, which at short inter-event intervals could only be measured following a template subtraction procedure, makes the determination of the exact kinetics a challenging task.

We found that recovery from postsynaptic spike depression could be well described by the sum of two exponential functions, which was in agreement with our earlier results (Lorteije et al., 2009). The sodium channels of principal neurons also recover biphasically, at similar kinetics as the recovery from AP depression (Leão et al., 2005), suggesting – similar to the presynaptic situation - a prominent role for the recovery from sodium channel inactivation in recovery from AP depression. Somatic sodium channel density is low in the MNTB principal neurons (Leão et al., 2005, 2008), suggesting changes in the axonal domain. Postsynaptic measurements were somatic, whereas the axonal and somatic AP can differ substantially (Scott et al., 2007; Yu et al., 2008). The rate of rise of the somatic AP is influenced by the backpropagation from the AIS. Moreover, the backpropagating AP will interact with the large, axosomatic calyceal input. These considerations make inferences about the mechanisms underlying these observed changes somewhat speculative, especially with regard to possible changes during multiple APs.

The voltage clamp recordings from principal neurons in MNTB slices did not provide any evidence for a change in the voltage-dependent recovery from sodium channel inactivation in the *Ube3a* mice, even though we cannot exclude that small effects were missed, since the voltage clamp in the whole cell recordings was suboptimal, recordings were performed at room temperature, and the slow component previously observed by Leão et al. (2005) was not observed by us. Interestingly, the kinetics of the recovery from inactivation of sodium channels or MNTB principal neurons were shown to be different in deaf mice, but the difference was in the slow component of recovery (Leão et al., 2006), which was not altered in the *Ube3a* mice.

The most likely mechanism underlying the faster recovery from AP depression in the *Ube3a* mice is related to the more negative resting potential, which based on our voltage clamp recordings (**Figure 9**) and previous results (Leão et al., 2005; Mathews, 2008) should speed up recovery from sodium channel inactivation considerably. However, we cannot exclude that the observed changes in AP recovery were mediated by potassium channels, especially since we observed shorter AP half widths in the *Ube3a* principal neurons. The calyx of Held synapse contains a multitude of different potassium channels (Johnston et al., 2010), and a subset of these show rapid facilitation (Yang et al., 2014), which could contribute to spike depression. A concomitant switch in the K channel composition of the AIS contributes to the increased excitability observed in the chick nucleus laminaris following cochlear ablation (Kuba et al., 2015).

### Increase in AIS Length

We saw about a 25% increase in AIS length of the principal neurons, somewhat larger than the increase observed previously in the hippocampus of *Ube3a* mice (Kaphzan et al., 2011). It is not known whether the observed increase in AIS length was accompanied by a change in its location, but in general, changes in AIS length, with corresponding changes in total sodium conductance, are more effective in regulating neuronal excitability than changes in location (Gulledge and Bravo, 2016). An increase in AIS length by itself may lead to shorter interspike intervals, a larger maximal rate of rise and more negative AP threshold (Kuba and Ohmori, 2009; Baalman et al., 2013). A longer AIS (with the resulting larger Na conductance) can help to overcome the electrical load of the soma and dendrites (Kuba et al., 2006; Baranauskas et al., 2013; Gulledge and Bravo, 2016; Hamada et al., 2016). The impact that a change in AIS length will have on neuronal excitability will also depend on changes in its localization and ion channel composition (Kuba et al., 2006, 2015; Baranauskas et al., 2013; Evans et al., 2015; Gulledge and Bravo, 2016; Hamada et al., 2016). A larger AIS may move spike initiation further away from the soma, making it less susceptible for the depolarization induced by calyceal EPSPs at the soma (Kuba et al., 2006; Scott et al., 2014). Even though some of the signal transduction mechanisms underlying AIS plasticity are beginning to be identified (Trunova et al., 2011; Galiano et al., 2012; Evans et al., 2013, 2015, 2017), how UBE3A contributes to the regulation of its length remains to be addressed.

Our results are largely consistent with a previous *in vitro* study showing a more negative resting membrane potential, larger AP amplitude and increased maximal rate of rise of the APs in CA1 pyramidal neurons of *Ube3a* mice (Kaphzan et al., 2011), but differ from a recent *in vivo* study in layer 2/3 pyramidal neurons of the visual cortex, in which increased intrinsic excitability in *Ube3a* mice was found to be mainly due to an increased membrane resistance (Wallace et al., 2017). The hippocampal and cortical pyramidal neurons are more similar to each other than to MNTB neurons, which are fast, glycinergic neurons that serve mostly a relay function. Why the differences in excitability observed in the present study are more similar to the changes observed in hippocampal neurons than in neocortical neurons is presently not clear.

### Homeostatic Plasticity or Gain-of-Function?

Evidence was presented that the more negative resting potential in hippocampal neurons likely result from increased expression of the α1 subunit of the Na/K-ATPase, and the changes in the AP from increased expression of Nav1.6 (Jiang et al., 1998; Kaphzan et al., 2011, 2013), and similar results have been obtained in the *Drosophila* neuromuscular junction (Valdez et al., 2015; Hope et al., 2017). The Nav1.6 subunit can be expected to be the dominant type of sodium channel in the young-adult MNTB (Leão et al., 2005). To explain the results in the hippocampus, it was suggested that the increase of the AIS length of *Ube3a* model mice might reflect a homeostatic response to a reduction in neuronal excitability, driven by the increase of α1-NaKA expression (Kaphzan et al., 2011, 2013). It has been well established that the AIS is a dynamic structure, and that the expression of sodium channels can depend on input activity (Grubb and Burrone, 2010; Kuba et al., 2010, 2014, 2015; Gutzmann et al., 2014; Nozari et al., 2017). It is remarkable that the increase in AIS length was associated with a more negative membrane potential, both at hippocampal CA1 pyramidal neurons (Kaphzan et al., 2011) and in the present study, since a chronic depolarization induces a rapid change in AIS length in cultured neurons (Evans et al., 2015). A homeostatic mechanism, as suggested as a compensatory mechanism for the changes in membrane potential observed in the hippocampus, seems insufficient to explain our results. A homeostatic mechanism would be expected to compensate for a decrease in excitability, thus keeping the reliability on average constant. However, we observed fewer postsynaptic failures in the *Ube3a* mutant mice in the absence of obvious changes in the input firing patterns to the principal neurons. This gain-of-function phenotype thus indicates that a simple homeostatic mechanism is insufficient to explain the observed results.

### Reduced STD

During auditory stimulation, complex effects were observed on short-term synaptic depression (STD) of the EPSP. In the post-hearing calyx of Held synapse, STD is presynaptic (Joshi and Wang, 2002; Taschenberger et al., 2002, 2005; Yamashita et al., 2003; Renden et al., 2005; Koike-Tani et al., 2008), even though it has not yet been confirmed that this also holds true for the young-adult mice that were studied here. In *Ube3a* animals, the sound-evoked EPSP showed less STD, and the depression level was largely independent of spontaneous activity, which contrasts with the *Drosophila* neuromuscular junction, where increased STD was observed (Valdez et al., 2015; Li et al., 2016). Most likely, the observed faster recovery from synaptic depression contributed to the reduction in STD we observed during tone presentation. We previously also observed faster recovery from STD in the *Cacna1a*^S218L^ mutant mouse model of Familial Hemiplegic Migraine (FHM; Di Guilmi et al., 2014). However, in the *Cacna1a*^S218L^ knock-in mice increased STD was observed, probably due to increased release probability of the readily releasable pool owing to elevated basal [Ca^2+^]_i_, indicating that there are essential differences in the presynaptic changes in the *Cacna1a*^S218L^ and the *Ube3a* mice.

## Outlook

Here we have provided the first *in vivo* characterization of the intrinsic membrane properties, synaptic transmission and AIS morphology of MNTB neurons in *Ube3a* mice. The enhanced synaptic transmission and elevated AP amplitude are likely to contribute to the hyperexcitability and increased seizure susceptibility in *Ube3a* mice and AS patients even though changes in GABAergic transmission are also an important factor in epileptogenesis (Judson et al., 2016). A homeostatic regulation of membrane excitability (Kaphzan et al., 2011, 2013) seems insufficient to explain the observed gain of function. How decreased UBE3A-mediated proteasomal degradation causes abnormalities in excitability and AIS structure deserves further study. The molecular mechanisms for the increased resistance to short-term synaptic depression in the calyx of Held synapse of *Ube3a* mice also remain to be identified. Identification of pre- and postsynaptic targets of UBE3A in the MNTB would be a helpful step toward these goals.

## Author Contributions

GvW and YE designed and generated the *Ube3a^E*113*X^* mice. GvW did the Western blot and immunocytochemical analysis of these mice. TW and JB performed and analyzed electrophysiological experiments. TW did morphological analysis. JB wrote analysis software. TW and JB wrote the manuscript. All authors contributed to the planning of the experiments, commented on earlier versions of the manuscript, and approved the final version of the manuscript.

## Conflict of Interest Statement

The authors declare that the research was conducted in the absence of any commercial or financial relationships that could be construed as a potential conflict of interest.
